# Autosomal Dominant Hyper‐IgE Syndrome Patients Retain IL10‐Producing preTh17‐Cells That Are Activated by Opportunistic Pathogens and Support IgE Production

**DOI:** 10.1111/all.70266

**Published:** 2026-02-25

**Authors:** Giorgia Moschetti, Chiara Vasco, Francesca Clemente, Paola Larghi, Sara Maioli, Edoardo Scarpa, Elena Carelli, Nadia Pulvirenti, Maria Lucia Sarnicola, Mariacristina Crosti, Manal Bel Imam, Willem van de Veen, Loris Rizzello, Sergio Abrignani, Lucia A. Baselli, Rosa Maria Dellepiane, Maria Carrabba, Giovanna Fabio, Jens Geginat

**Affiliations:** ^1^ INGM, Istituto Nazionale Genetica Molecolare “Romeo ed Enrica Invernizzi” Milan Italy; ^2^ Department of Clinical Sciences and Community Health Università degli Studi di Milano Milan Italy; ^3^ Department of Pharmaceutical Sciences Università degli Studi di Milano Milan Italy; ^4^ Swiss Institute of Allergy and Asthma Research Davos Switzerland; ^5^ Fondazione IRCCS Ca' Granda Ospedale Maggiore Policlinico Pediatric Immunorheumatology Unit, ERN‐RITA Center Milan Italy; ^6^ Department of Internal Medicine Fondazione IRCCS Ca' Granda Ospedale Maggiore Policlinico, ERN‐RITA Center Milan Italy

**Keywords:** IgE, immune deficiencies, interleukins, T cells

## Abstract

**Background:**

Autosomal Dominant‐Hyper‐IgE Syndrome (AD‐HIES) is caused by dominant‐negative (DN) STAT3 mutations and characterized by high IgE levels, a lack of Th17‐cells, and recurrent infections with extracellular pathogens. We previously identified an enigmatic population of IL‐10 producing CCR6^+^B‐helper T‐cells and investigated here their relationship to Th17‐cells and STAT3 signaling requirements.

**Methods:**

Human blood lymphocytes were analyzed by multiparametric flow cytometry in healthy donors and AD‐HIES patients. Analysis was performed by conventional gating or with bioinformatic tools. FACS‐purified T‐cell subsets were activated in vitro and Th17 differentiation assessed. T‐cell antigen specificities were assessed by activation with heat‐killed pathogens or antigenic peptide pools. B helper capacities were determined according to antibody secretion in B‐T co‐cultures by ELISA.

**Results:**

CCR6^+^Th‐cells that lacked subset‐defining differentiation markers (“CCR6^SP^”) were mostly non‐polarized central memory T‐cells (T_CM_) that produced IL‐10 and expressed RORγt. They were pre‐committed to a Th17 fate, since TCR stimulation in the absence of polarizing cytokines induced efficient Th17 differentiation. The latter was promoted by an autocrine loop of STAT3‐activating cytokines. CCR6^+^Th‐cells were reduced in patients with DN‐STAT3 mutations but contained activated CCR6^SP^T‐cells that produced IL‐10 and responded vigorously to AD‐HIES‐associated pathogens. These residual CCR6^+^Th‐cells provided B‐cell help for IgG and IgE production.

**Conclusions:**

Th17 differentiation in AD‐HIES patients was not completely impaired but arrested at an intermediate stage of IL‐10‐producing “pre‐Th17”‐cells. Surprisingly, DN‐STAT3 mutations did not inhibit IL‐10 production by CD4^+^T‐cells. Pre‐Th17‐cells were activated by AD‐HIES‐associated pathogens and possessed B‐helper functions, suggesting that they are not protective but may promote aberrant IgE production.

## Graphical abstract

IL‐10 producing CCR6^+^Th‐cells are central memory T‐cells that express ROR‐γt and differentiate to Th17‐cells via an autocrine loop of STAT3‐activating cytokines (preTh17). STAT3‐deficient AD‐HIES patients lack Th17‐ and Tfh17‐cells but retain preTh17‐ and Th1/17‐cells. preTh17‐cells in AD‐HIES patients are activated by opportunistic pathogens to produce IL‐10 and support IgE production.

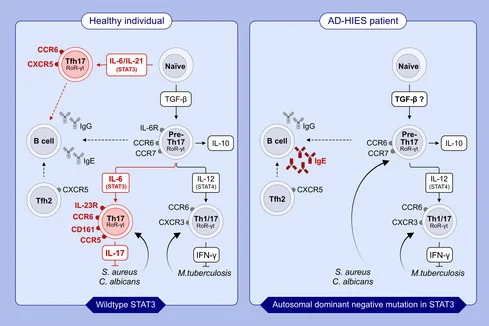

**Abbreviations**
AD‐HIES: autosomal dominant hyper‐IgE syndrome

*C. albicans*

:
*Candida albicans*

CCR: C‐C motif chemokine receptor
CXCR: C‐X‐C motif chemokine receptor
IFN‐γ: interferon gamma
IL: interleukin

*M. tuberculosis*

: *Mycobacterium tuberculosis*
ROR‐γt: RAR‐related orphan receptor‐gamma t

*S. aureus*

:
*Staphylococcus aureus*

STAT: signal transducer and activator of transcription
Tfh: T follicular helper cell
TGF‐β: transforming growth factor beta
Th: T helper cell

## 1 Introduction

CD4^+^T‐cells play a pivotal role to coordinate adaptive immune responses and acquire specialized functions upon priming by different classes of pathogens. Initially, Th1‐cells that fight intracellular pathogens and Th2‐cells that are important to contain extracellular parasites have been described [1]. Later, Th17‐cells and Tfh‐cells were identified that control infections with extracellular bacteria or fungi and promote B‐cell antibody responses, respectively. Th17‐cells express the transcription factor ROR‐γt, the chemokine receptor CCR6, the IL‐23R and produce IL‐17, IL‐22 [2, 3] and GM‐CSF [4]. They are highly heterogeneous [1, 5, 6, 7]. In humans, four CCR6^+^Th subsets with characteristic cytokine profiles and functions have been classified mainly according to chemokine receptor expression, namely CCR5^+^/CD161^+^Th17‐cells [8, 9], CCR10^+^Th22‐cells [10, 11]. CXCR3^+^Th1/17‐cells [12, 13, 14] and CXCR5^+^Tfh17‐cells [15]. We previously identified an additional CCR6^+^Th‐cell subset that lacked these subset‐defining surface markers called “CCR6^single‐positiv(SP)”^T‐cells. They were largely non‐polarized, but produced IL‐10 [16] and possessed B‐helper functions [17]. How this enigmatic subset is related to the established CCR6^+^Th subsets is unclear. Th17 and Tfh differentiation, as well as IL‐10 production, are promoted by cytokines that signal via STAT3, namely IL‐6 [18], IL‐21 [19, 20] and IL‐10 [21]. However, if IL‐10 producing CCR6^SP^T‐cells are also STAT3‐dependent it unknown.

The in vivo relevance of STAT3 in human T‐cell differentiation is evident in patients with autosomal dominant Hyper‐IgE Syndrome (AD‐HIES), which contain dominant‐negative (DN) mutations of STAT3 [22, 23]. Deficient STAT3 signaling and its broad expression explain the multisystem manifestations of AD‐HIES syndrome including dermatitis, pulmonary disease, vasculopathy, and skeletal and connective tissue abnormalities. High levels of serum IgE, eczema, and recurrent infections are the hallmarks of AD‐HIES. Severe bacterial infection, cold abscess, osteomyelitis, chronic mucocandidiasis, and invasive fungal infections are common [24]. The recurrent infections in AD‐HIES are explained by the lack of IL‐17‐producing T‐cells [25, 26].

Here we show that CCR6^SP^T‐cells are central memory T‐cells (T_CM_) that are pre‐committed to a Th17 fate (pre‐Th17). Surprisingly, they are generated in STAT3‐deficient AD‐HIES patients, are activated by AD‐HIES‐associated pathogens to produce IL‐10 and maintain B‐helper functions.

## 2 Material and Methods

### 2.1 Patients

AD‐HIES was diagnosed according to the 2014 protocol by the Italian Primary Immunodeficiency Network (IPINet) group of the Italian Association of Pediatric Hematology Oncology (AIEOP) and patients of this study are part of the Italian AD‐HIES cohort [27]. All patients carried a pathogenic DN mutation of STAT3. Genetics and clinical features of patients are shown in Figure 3A. All patients are followed according to the IPINet protocol. The local ethics committee approved this study (1097_2019bis, protocol ID #1305) and informed consent was obtained from all patients. This study adhered to the ethical principles outlined in the Declaration of Helsinki and followed the guidelines of Good Clinical Practice.

### 2.2 Flow Cytometry

Human peripheral blood mononuclear cells (PBMCs) were isolated by standard Ficoll–Hypaque density gradient centrifugation. PBMCs were stained with a combination of fluorochrome‐conjugated antibodies as specified in Table S1. Acquisition of stained cells was performed with a FACSymphony (Becton Dickinson, BD) and data analyzed with the FlowJo software (BD, version 10.8.1). To assess cytokine producing capacities, CD4^+^T‐cell subsets were sorted on a FACSAria III (BD) and stimulated with 50 ng/mL phorbol 12‐myristate 13‐acetate (PMA) and 500 ng/mL ionomycin for 4 h in the presence of 10 μg/mL Brefeldin A (all from Sigma Aldrich) in the last 2 h. Cells were incubated with the Intracellular Fixation & Permeabilization Buffer Set (Thermo Fisher) and stained for intracellular cytokines (Table S1). IL‐10 production was also assessed in FACS‐purified T‐cell subsets following 30 h stimulation with immobilized anti‐CD3 antibodies (5 μg/mL; Biolegend) and 10 ng/mL IL‐2 (Miltenyi) with an IL‐10 secretion assay (Miltenyi), and after 18 h of stimulation of PBMCs with Staphylococcal enterotoxin B (SEB). Cytokine receptor expression was assessed following stimulation with 10 ng/mL IL‐6 or IL‐23 (Miltenyi) at 37°C for 30 min. Cells were stained with anti phospho‐STAT antibodies to assess STAT phosphorylation [19, 28].

### 2.3 Immunofluorescence and Imaging

IL‐10 expression was analyzed in co‐cultures of sorted CCR6^+^T‐cell subsets, autologous monocytes and SEB. Experimental details of immunofluorescence are provided in the Supporting Information Material and Methods section.‐Fluorescence images were acquired using an inverted STELLARIS 8 Confocal Microscope (Leica Microsystems) equipped with a 405 laser and a tuneable pulsed white light laser, utilizing a 40× air objective (numerical aperture [NA] 0.95) in three independent experiments. Quantification was performed using NIS‐Elements V.5.30 software (Lim‐Instruments). Ad‐hoc designed pipelines of digital imaging analysis were implemented using the General Analysis 3 module of NIS‐Elements to determine the intensity of fluorescence associated with the single cells present in the images.

### 2.4 Th17 Differentiation

CCR6^+^ and CCR6^−^T‐cell subsets were sorted according to surface receptors as reported [17]. To obtain non‐polarized cells, T‐cell subsets were stimulated with PMA and ionomycin, a cytokine secretion assay performed, and IL‐17A^−^IFN‐γ^−^ or IL‐17^−^IL‐22^−^ cells sorted. Cells were stimulated with anti‐CD3/28 beads (Invitrogen) at a 1:1 ratio. Recombinant cytokines (TGF‐β, IL‐4, IL‐6, IL‐12, IL‐21 (R&D) or WNT10a, (Peprotech)) were added at 10 ng/mL, antagonists of cytokines or cytokine receptors at 10 μg/mL (anti‐IL‐10, anti‐IL‐6 (Biolegend)) or 1 μg/mL (anti‐IL‐10R and sIL‐21R (R&D)). After 6 days, cells were re‐stimulated with PMA and ionomycin and analyzed by cytokine staining. IL‐6 production was measured in 6d supernatants of anti‐CD3/28‐stimulated T‐cells by ELISA assay (Tebu Bio).

### 2.5 Bioinformatic Analysis

Flow cytometry data were imported into FlowJo software (version 10.8.0) and a random down‐sample of 6000 events for CD4^+^ T‐cells was performed. The FCS file was analyzed by the R software. Sample batches were read using read.flowSet (2.6.0) from the flowCore R package. We applied the Logicle transformation that allows multiple samples to estimate transformation parameters. To reduce batch effects, we normalized the signal of each marker with the function gaussNorm from the flowStat package (4.6.0). Samples were concatenated into a SingleCellExperiment object in R using the function prepData from the CATALYST R package. We performed high‐resolution, unsupervised clustering and meta‐clustering using FlowSOM (2.2.0) and ConsensusClusterPlus (1.58.0) package [29]. Manually annotated clusters were subsequently visualized in UMAPs. Functional pseudo‐time analysis to infer the differentiation trajectory of cells was carried out by Diffusion Maps using the function run Diffusion Map on the SingleCellExperiment object using default parameters.

### 2.6 Antigen Specificity Assay

Stimulation with heat‐killed pathogens or pathogen‐derived peptide pools was performed for 3 h with 10^6^ PBMCs in RPMI 1640 complete medium supplemented with 5% human serum. Monensin (BD GolgiStop, BD Biosciences) was added for an additional 15 h of incubation, and cells were analyzed for the co‐expression of CD69 and cytokines. Heat‐killed pathogens and antigenic peptide pools are listed in Table S2.

### 2.7 B Helper Assay

Naïve, CCR6^+^ and CXCR5^+^CD4^+^T‐cells were isolated from total PBMCs of AD‐HIES patients or healthy donors by FACS sorting (FACSAria III, BD). T‐cells were co‐cultured in V‐shaped 96‐well plates with autologous CD20^+^B‐cells at a 1:1 ratio in RPMI supplemented with 10% serum (Euroclone), 200 U/mL IL‐2, and Staphylococcal Enterotoxin B (SEB 100 ng/mL Merck). At day 6‐7, co‐cultures were supplemented with fresh medium and supernatants were harvested at day 14. Supernatants were tested for total IgG production by ELISA (Biolegend, Thermofisher Scientific, Abcam). To analyze IgE production, B‐T co‐cultures were performed with CXCR3^−^B helper subsets at a 3:1 B‐T ratio with 1 μg/mL SEB in the presence of 200 U/mL IL‐2 and 10 ng/mL IL‐4. Supernatants were tested by sandwich ELISAs for the secretion of IgG (BD) and IgE (Capture antibody: BD; Detection antibody: Invitrogen; Standard: Abcam).

### 2.8 Statistical Analysis

Statistics were performed with the Prism software (version 7; GraphPad Software Inc., La Jolla, CA, USA). Statistical significance was evaluated using a student's *t*‐test for the comparison of two groups with Mann–Whitney's or Welch's correction to analyze variables that were not normally distributed or by One way‐ANOVA for comparison of more than two groups (Tukey's post hoc correction). Significances were reported as *p < 0.05, **p < 0.01, ***p < 0.001. Error bars report ±SEM in all Figures.

## 3 Results

### 3.1 CCR6^SP^T‐Cells Are Central Memory T‐Cells (T_CM_ ) That Express ROR‐γt and Produce IL‐10

We previously identified an enigmatic population of CCR6^+^ B‐helper T‐cells that lacked gene signatures and established surface markers of Th17‐, Th1/17‐, and Tfh17‐cells called CCR6^“single‐positive”(SP)^T‐cells [17]. To map the differentiation stage of CCR6^SP^T‐cells, we first performed a deep phenotypic analysis in the blood of healthy donors (Figure 1A; Figure S1A). CCR6^SP^T‐cells expressed low levels of the gut‐homing receptors CCR9 and β7‐integrin and lacked CXCR6. They expressed, however, several receptors involved in skin‐homing, including CCR10, CLA, β1‐integrin, and CCR4 (Figure 1A; Figure S1B). Since human CCR6^+^CCR10^+^T‐cells contain Th22‐cells [1, 10, 11] we analyzed them separately and focused the further analysis on CCR10^−^CCR6^SP^‐T‐cells (Figure S1A). Most CCR6^SP^T‐cells displayed a T_CM_ phenotype since they co‐expressed CCR7 and CD62L (Figure 1B). They expressed also high levels of CD27 and lacked PD1 (Figure S1C). Moreover, they expressed TCF7 and LEF1 (Figure 1C), transcription factors of the WNT pathway that are progressively lost upon T‐cell differentiation (Figure S1D) and promote stemness. Expression of ROR‐γt, the lineage‐defining transcription factor of Th17‐cells, was, as expected, restricted to CCR6^+^T‐cells (Figure S1E). Among CCR6^+^T‐cell subsets, it was expressed at a high level in Th17‐cells and Th1/17‐cells. Importantly, however, ROR‐γt was also clearly detectable in CCR6^SP^T‐cells (Figure 1D).

**FIGURE 1:**
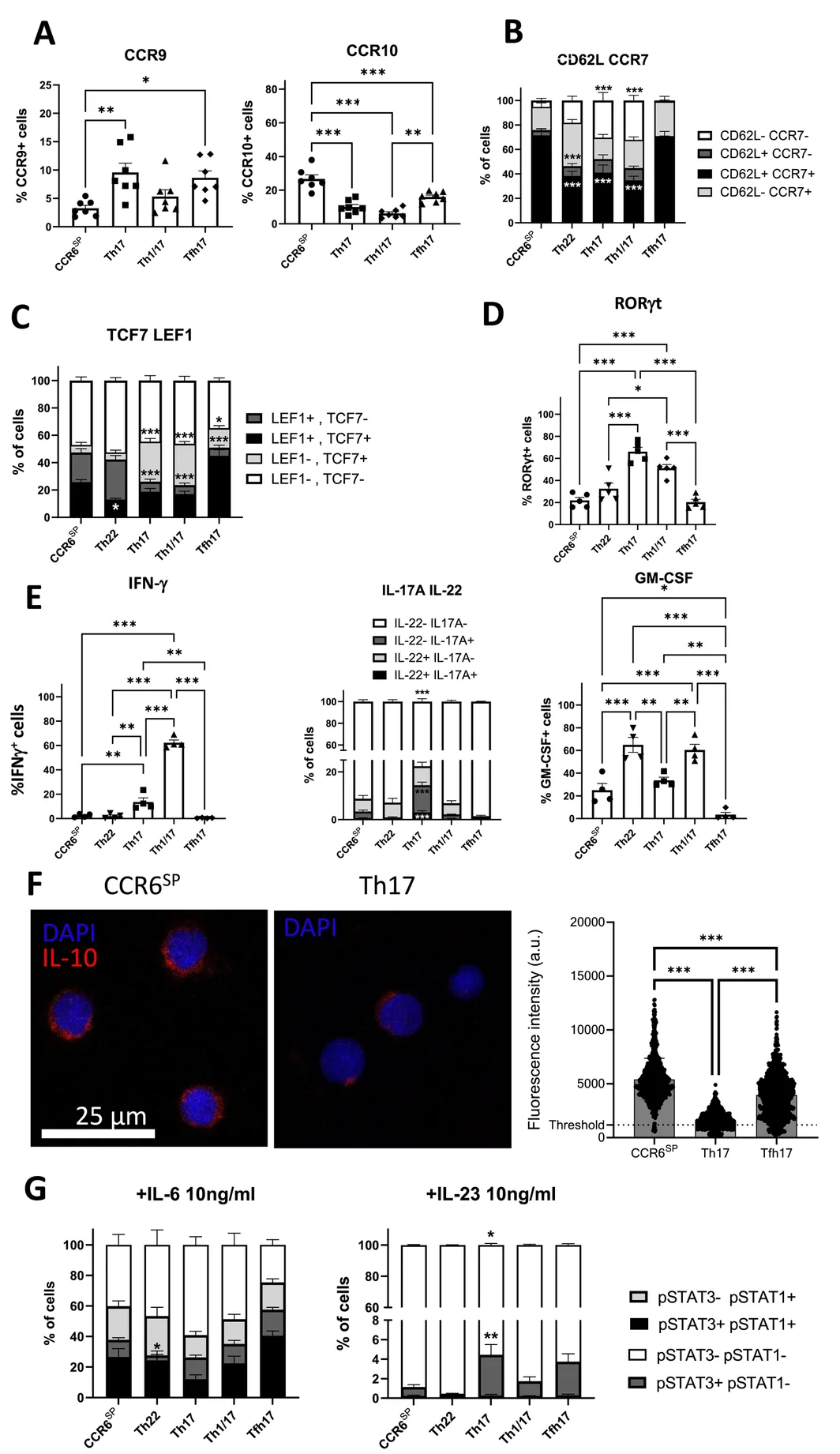
CCR6^SP^‐T‐cells are T_CM_ that express ROR‐γt and produce IL‐10. (A) Ex vivo frequencies of CCR9‐ or CCR10‐expressing cells among gated CD4^+^CCR6^+^Th‐cell subsets (see gating strategy in Figure S1A) in healthy donors (*n* = 7) Expression of (B) CD62L and/or CCR7 (stacked histograms, *n* = 7; Th22: CCR6^+^CCR10^+^). (C) TCF7 and/or LEF1 (*n* = 7) and (D) RORC/γt in healthy donors (*n* = 5). (E) Production of IFN‐γ, IL‐17 and/or IL‐22 and GM‐CSF following 4 h stimulation of the indicated FACS‐purified CCR6^+^T‐cell subsets from healthy donors with PMA and Ionomycin (*n* = 4). (F) IL‐10 production by the indicated CCR6^+^T‐cell subsets from healthy donors upon stimulation with monocytes and SEB for 16 h was analyzed by immunofluorescence. At least 1600 cells from 3 different donors were analyzed. (G) Expression of functional cytokine receptors in T‐cell subsets from healthy donors was assessed after stimulation for 30 min with the relevant cytokines by STAT phosphorylation. Statistically significant differences were calculated by One‐way ANOVA and reported as *p < 0.05, **p < 0.01, ***p < 0.001. */*** on the top of stacked histograms report significant differences of double‐negative cells.

We then assessed cytokine producing capacities of CCR6^+^T‐cell subsets upon ex vivo stimulation with PMA and Ionomycin (Figure 1E). Th17‐, Th1/17‐, and Th22‐cells produced, as expected, preferentially IL‐17, IFN‐γ, and IL‐22, respectively [10, 12, 15]. Th1/17‐ and Th22‐cells produced in addition high levels of GM‐CSF. CCR6^SP^T‐cells and Tfh17‐cells produced only low levels of these effector cytokines, suggesting that they were predominantly non‐polarized cells. Since PMA plus Ionomycin stimulation is suboptimal to induce IL‐10 production in human CCR6^+^Th‐cells [16], we analyzed IL‐10 production in CCR6^+^T‐cell subsets stimulated with monocytes and the superantigen SEB by immunofluorescence (Figure 1F; Figure S1F). IL‐10 was produced at significantly higher levels by CCR6^SP^‐T‐cells as compared to Tfh17‐cells and Th17‐cells. Finally, since IL‐6R and IL‐23R promote, respectively, early and late Th17 differentiation [8, 18, 30, 31] we assessed their expression according to ligand‐induced STAT phosphorylation [19] (Figure 1G; Figure S1G). IL‐6 induced STAT phosphorylation in most CCR6^SP^T‐cells, whereas IL‐23 largely failed to do so.

In summary, CCR6^SP^T‐cells are largely non‐polarized, IL‐10‐producing T_CM_ that express low levels of ROR‐γt but lack the IL‐23R, suggesting that they are at an early stage of Th17 differentiation.

### 3.2 An Autocrine Loop of STAT3‐Activating Cytokines Promotes TCR‐Induced Th17 Differentiation of CCR6^SP^T‐Cells in the Absence of Polarizing Cytokines

Since CCR6^SP^T‐cells expressed ROR‐γt we investigated if they were pre‐committed to a Th17 fate. To exclude contributions of contaminating effector T‐cells, we purified non‐polarized T‐cells by depleting cells that could produce IL‐17, IFN‐γ, or IL‐22 ex vivo with a cytokine secretion assay (Figure S2A). The capacity to produce the depleted cytokines was then analyzed after in vitro culture in the absence of Th17‐polarizing cytokines for 7 days. CCR6^SP^T‐cells acquired high levels of IL‐17 and IL‐22 producing capacities under these neutral conditions, whereas CCR6^−^ control Th‐cells did not (Figure 2A). Notably, CCR6^SP^T‐cell produced also significantly higher amounts of IL‐17 as compared to Tfh17 and Th1/17‐cells (Figure 2B). To analyze the requirements of this spontaneous Th17 differentiation, we added cytokines that promote Th17 differentiation or alternative T‐cell fates. Acquisition of IL‐17 and IL‐22 producing capacities by CCR6^SP^T‐cells was weakly inhibited by TGF‐β and WNT10A (Figure 2C; Figure S2B), which activates LEF1/TCF7 [32]. Conversely, IL‐4 strongly inhibited Th17 differentiation and IFN‐γ production. IL‐12 had a weak inhibitory effect on IL‐17 but strongly induced IFN‐γ. Consequently, it promoted co‐production of IFN‐γ and IL‐17, as is characteristic for Th1/17‐cells [12, 19, 33] (Figure 2C; Figure S2C). We next investigated if cytokines that are produced by TCR‐activated CCR6^SP^T‐cells and activate STAT3, such as IL‐6 (Figure S2C), IL‐10 (Figure 1F) [17] and IL‐21 [19] could induce Th17 differentiation of CCR6^SP^T‐cells. CCR6^SP^T‐cells activated by anti‐CD3 and anti‐CD28 coated beads contained indeed high levels of phosphorylated STAT3 in the absence of exogenous cytokines (Figure 2D). Moreover, STAT3 phosphorylation was significantly inhibited when IL‐6, IL‐10 and IL‐21 were neutralized (Figure 2D). Importantly, neutralization of these STAT3‐activating cytokines also significantly reduced IL‐17 and IL‐22 production (Figure 2D; Figure S2D). Finally, addition of recombinant IL‐6 had a weak positive effect on Th17 differentiation that failed to reach statistical significance, whereas exogenous IL‐21 had no positive effect at all (Figure 2E).

**FIGURE 2:**
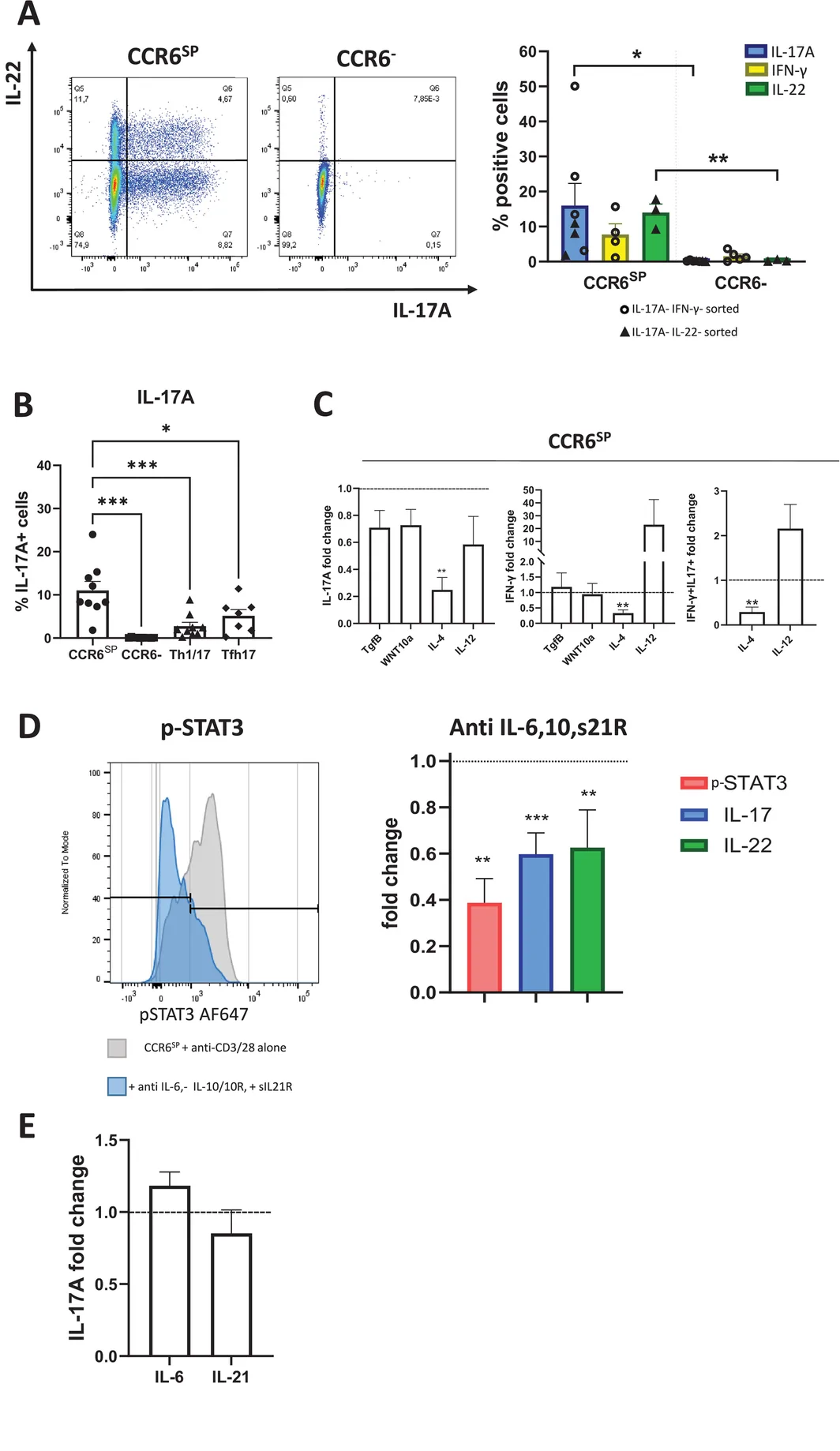
TCR‐activated CCR6^SP^T‐cells differentiate spontaneously to Th17‐cells. (A) FACS‐purified CCR6^SP^ T‐cells or CCR6^−^ control cells from healthy donors were depleted from residual IL‐17 and IFN‐γ (*n* = 4) or IL‐17 and IL‐22 producing cells (*n* = 3) with a cytokine secretion assay (see Figure S2A). Cells were activated with anti‐CD3/28‐coated beads and the induction of the previously depleted cytokines by PMA plus Ionomycin was analyzed by intracellular staining. Left: Dot plots of IL‐17 and IL‐22 induction in one representative donor. Right: Bars report the frequencies of cytokine‐producing cells. IL‐17^+^ and IFN‐γ^+^‐depleted cells are indicated by filled circles, IL‐17^+^ and IL‐22^+^ depleted cells by triangles. Statistical significance for each cytokine was calculated with a paired student's *t*‐test. (B) IL‐17 production by the indicated T‐cell subsets cultured for 6 days with anti‐CD3/28‐coated beads induced by PMA and Ionomycin. Statistical significance was calculated with One‐way ANOVA. (**C**) FACS‐purified total CCR6^SP^T‐cells from healthy donors were cultured for 6 days with anti‐CD3/28‐coated beads in the absence or presence of the indicated agonists and were analyzed for IL‐17 and IFN‐γ producing capacities. (D) FACS‐purified CCR6^SP^T‐cells from healthy donors were cultured with anti‐CD3/28‐coated beads in the absence or presence of antagonists of IL‐6, IL‐10 and IL‐21 and analyzed for STAT3 phosphorylation after 24 h or for IL‐17 and IL‐22 production after 6 days following brief restimulation with PMA and Ionomycin. (E) CCR6^SP^T‐cells were cultured with anti‐CD3/28‐coated beads in the absence or presence of recombinant IL‐6 or IL‐21. (C**–**E) Shown is the fold‐change as compared to CCR6^SP^T‐cells stimulated with anti‐CD3/28 beads alone (indicated by dotted lines). Statistical significance for each condition was calculated with a paired student's *t*‐test and is indicated by */**/***/**** on the top of the columns.

In conclusion, CCR6^SP^T‐cells produce cytokines upon activation that signal via STAT3 and promote autocrine Th17 differentiation. However, CCR6^SP^T‐cells also retain a relevant degree of plasticity [5, 34] and could still be instructed to become Th1/17‐cells.

### 3.3 Residual CCR6 ^+^T‐Cells in AD‐HIES Contain Th1/17‐ and CCR6^SP^T‐Cells and Produce IL‐10

To address the role of STAT3 in CCR6^SP^T‐cells in vivo, we then analyzed CD4^+^T‐cells from AD‐HIES patients (Figure 3A) [35]. CD4^+^T‐cells from AD‐HES patients were compared to healthy donors (HDs) by multi‐dimensional flow cytometry. Unsupervised principal component analysis (PCA) revealed significant differences (Figure 3B). Unified Manifold Approximation and Projection (UMAP, Figure 3C; Figure S3A–C) identified six CCR6^+^Th‐cell clusters, which were all reduced in AD‐HIES. Notably, cluster 9 contained CCR6^SP^T‐cells and co‐clustered with Th1/17_CM_ (Cluster 7) and Tfh (Cluster 8), but not with CCR6^+^CCR10^+^ clusters (Clusters 11 and 13, Figure S3A). The reduction of CCR6^+^Th clusters was compensated by an increase of naïve T‐cells (Cluster 1, Figure 3D), whereas Th1 clusters (Clusters 6, 16) were unaffected. Diffusion map analysis suggested a progressive differentiation from naïve to effector T‐cells, with T_CM_ and T_EM_ being respectively early and late differentiation intermediates (Figure 3E). Moreover, the diffusion map was consistent with an early‐to‐intermediate differentiation stage of CCR6^SP^T‐cells, positioning them between T_FH_‐ and other T_CM_ clusters.

**FIGURE 3:**
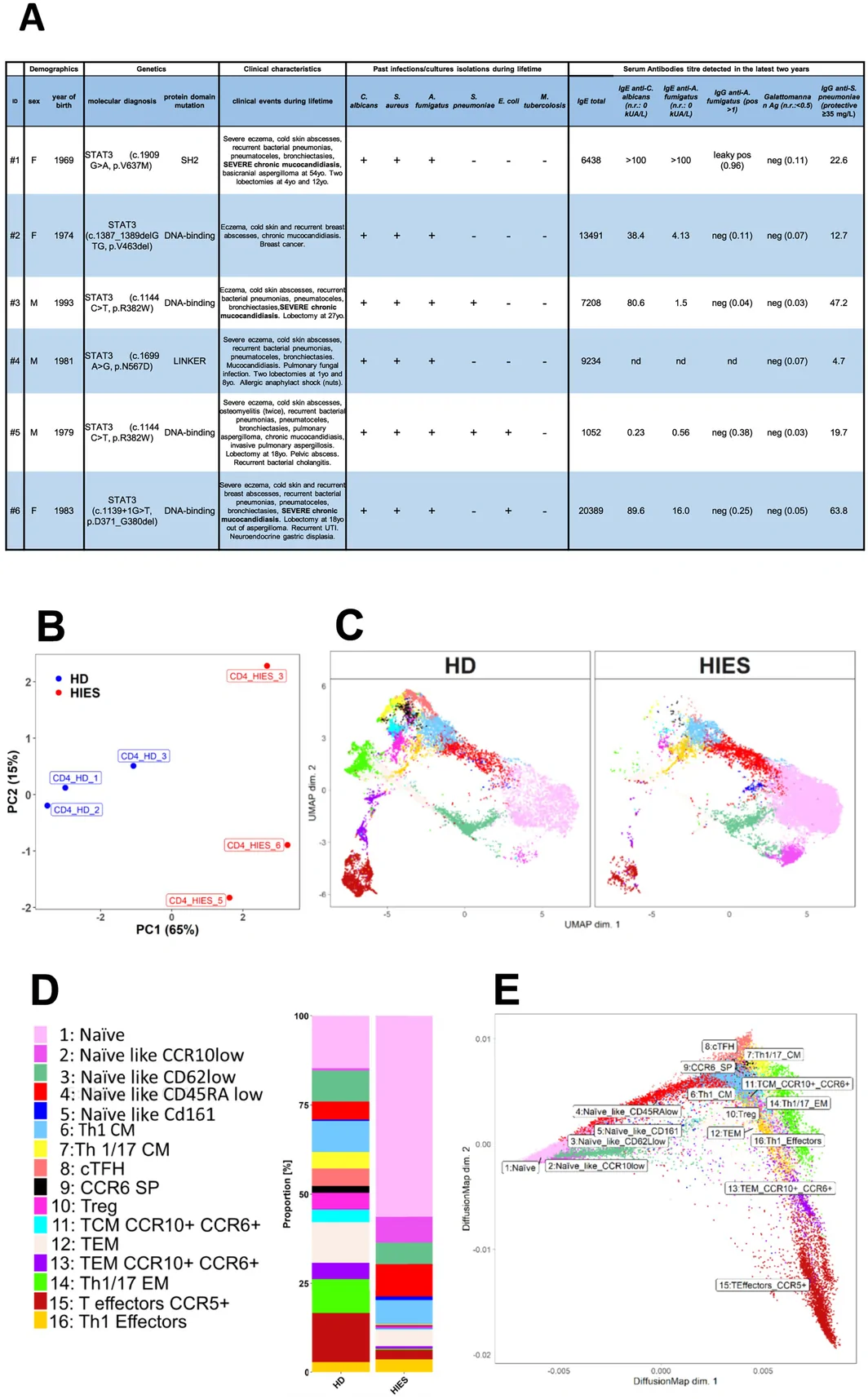
Unbiased analysis of the CD4^+^T‐cell compartments unveils a selective decrease of CCR6^+^T‐cell clusters in AD‐HIES. (A) Table showing mutations and clinical features of AD‐HIES patients, including total and fungi‐specific IgE levels. (B) Principal Component Analysis of 3 AD‐HIES patients and 3 healthy donors (HDs), respectively colored in blue and in red. Number in the label indicated the sample IDs. (C) UMAP maps of the 16 identified CD4^+^T‐cell clusters marked with different colors in the blood of HD and AD‐HIES patients. (D) Bar‐plots showing cluster distributions in the blood of HD and AD‐HIES patients. (E) Diffusion Map analysis of predicted cell differentiation pathways of the 16 identified CD4 T‐cell clusters in pseudo time. (C‐D) Color code and names of the 16 clusters are indicated in the Figure.

Manual gating confirmed that AD‐HIES patients exhibit an increase of naïve T‐cells (Figure S4A) and a reduction of all CCR6^+^T‐cell subsets (Figure 4A). Conversely, the frequencies of Th1, Th2 and of regulatory T‐cell subsets, i.e., FOXP3^+^Tregs and EOMES^+^Tr1‐like cells [36, 37], were similar (Figure S4B). Notably, while Th17‐ and Tfh17 subsets were largely undetectable in AD‐HIES, Th1/17‐cells and CCR6^SP^ were only partially reduced (Figure 4A). Consequently, when the relative contributions of subsets to CXCR3^−^CCR6^+^Th‐cells were analyzed, most cells in AD‐HIES patients displayed a CCR6^SP^ phenotype (Figure 4A). Consistently, CCR6^+^T‐cells from AD‐HIES patients contained higher frequencies of T_CM_ (Figure 4B), expressed higher levels of LEF1/TCF7 (Figure 4C) and lower levels of ROR‐γt (Figure 4D). Moreover, CCR6^+^T‐cells from AD‐HIES patients expressed significant lower levels of β1‐Integrin, but in mean higher levels of CCR9 (Figure 4E). The expression of other homing receptors was not significantly different (Figure S4C). Finally, CCR6^+^T‐cells in AD‐HIES lacked IL‐17 producing capacities, whereas production of IL‐21 (Figure 4F), IFN‐γ, IL‐2, IL‐4, TNF and GM‐CSF were similar (Figure S4D). They produced however significantly higher levels of IL‐10 (Figure 4F). This was also true upon more physiological TCR stimulation with anti‐CD3 antibodies [16] or with the superantigen SEB [38] (Figure S4E). Conversely, IL‐10 production by CCR6^−^Th‐cells remained overall low. Interestingly, also regulatory T‐cell subsets from AD‐HIES patients contained higher frequencies of IL‐10 producing cells (Figure S4F), and this increase reached statistical significance for EOMES^+^Tr1‐like cells [37] (Figure 4G).

**FIGURE 4:**
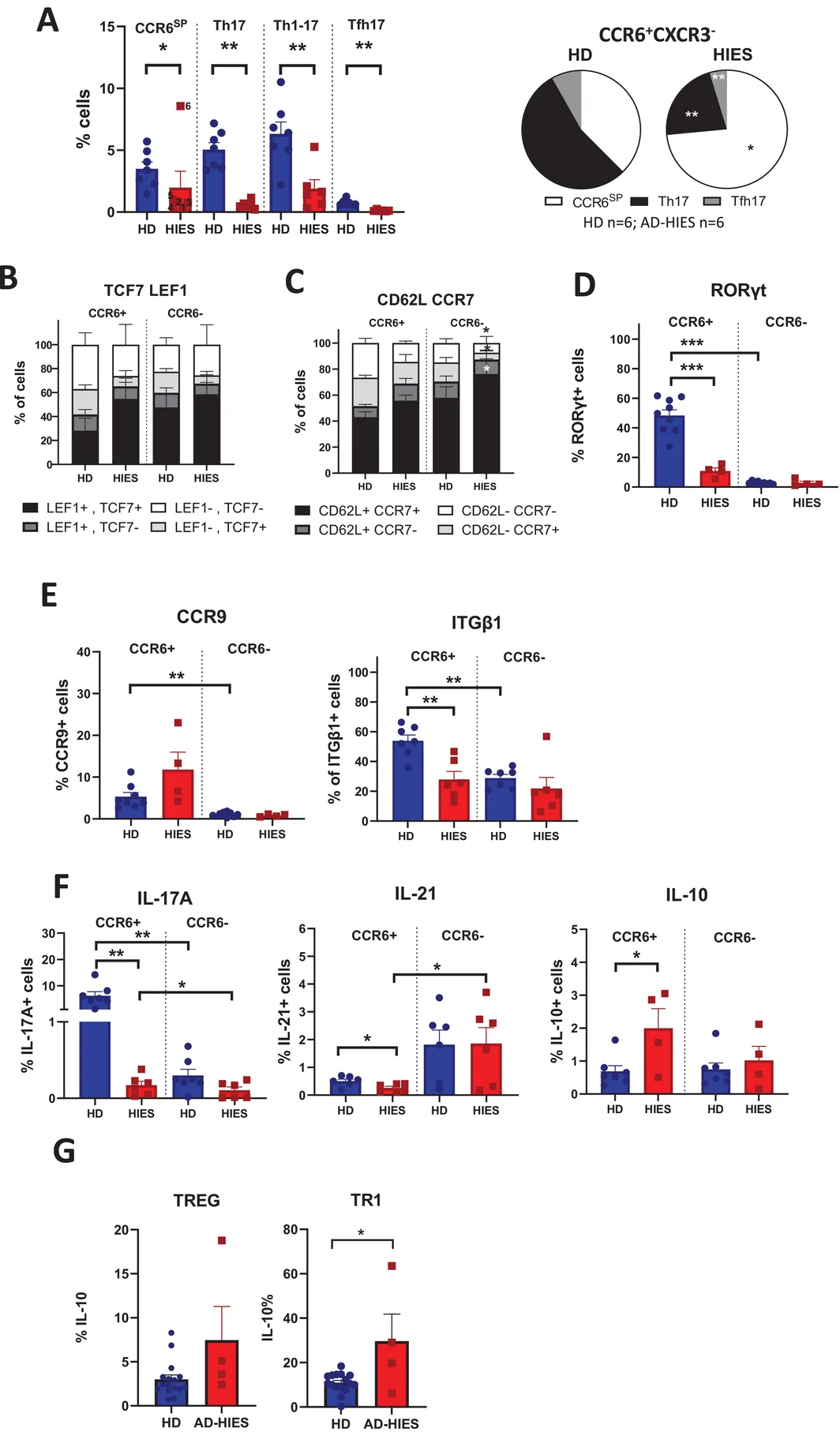
Residual CCR6^+^T‐cells in AD‐HIES patients display features of CCR6^SP^T‐cells and produce increased amounts of IL‐10. (A) Left: Frequencies of CCR6^SP^, Th17, Th1/17 and Tfh17 T‐cells among CD4^+^T‐cells in healthy donors (HD, blue bars, *n* = 7) and AD‐HIES patients (red bars, *n* = 6). The patient numbers of Figure 3A are indicated for CCR6^SP^T‐cells. Right: Pie charts show the relative contributions of phenotypical Tfh17‐cells, Th17‐cells and CCR6^SP^T‐cells to CXCR3^−^CCR6^+^T‐cells in the same cohorts. (B–E) Gated CCR6^+^ and CCR6^−^CD4^+^T‐cells in HD (*n* = 5–9) and AD‐HIES patients (*n* = 4–6) were analyzed ex vivo by flow cytometry for the expression of (B) CD62L and/or CCR7 (C) LEF1 and/or TCF7 (D) RORγt and (E) CCR9 and β1‐integrin. (F) Frequencies of IL‐17A, IL‐21 and IL‐10 expressing cells among CCR6^+^ and CCR6^−^T‐cells stimulated ex vivo with PMA and Ionomycin obtained from AD‐HIES patients (red bars, *n* = 4–6) and HD (blue bars, *n* = 7). (B–E) Statistical significance was calculated between HDs and AD‐HIES patients and between CCR6^+^ and CCR6^−^ subsets of the same individuals using unpaired or paired *t*‐tests, respectively. (F) Frequencies of IL‐10 expressing cells among Tregs (FOXP3^+^, left) and EOMES^+^Tr1‐like cells (GZMK^+^CCR6^−^ [36], right) from AD‐HIES patients (red bars, *n* = 4) and HD (blue bars, *n* = 16).

In conclusion, CCR6^+^Th‐cells in AD‐HIES patients are reduced, but not absent, and contain both IL‐10‐producing CCR6^SP^T‐cells and Th1/17‐cells.

### 3.4 CCR6 ^+^T‐Cells in AD‐HIES Are Activated by Opportunistic Pathogens to Produce IL‐10

CCR6^+^T‐cells from AD‐HIES patients were activated in vivo, as indicated by increased expression of PD1, CD69 and of the proliferation marker Ki67 (Figure 5A). We investigated if they might be activated by AD‐HIES‐associated infections. Notably, all analyzed patients had recurrent infections with *Staphylococcus aureus* , *Candida albicans* and *Aspergillus fumigatus* (Figure 3A). Moreover, some patients experienced infections with *Escherichia coli* and/or *Streptococcus pneumoniae* , whereas infections with *Mycobacterium tuberculosis* were never diagnosed. We could detect IL‐17 production by CCR6^+^T‐cells in response to all these heat‐killed pathogens in healthy donors, but not in AD‐HIES patients (Figure 5B). However, CCR6^+^T‐cells from AD‐HIES patients produced increased amounts of IL‐10 that reached statistical significance for most pathogens. CCR6^+^T‐cells from AD‐HIES patients produced also significantly more IL‐2 and IFN‐γ in response to *Escherichia coli* [8], *Streptococcus pneumoniae* and *Mycobacterium tuberculosis* [12] (Figure 5B). Conversely, CCR6^−^ control T‐cells responded poorly (Figure S5A). To control for possible bystander T‐cell activation by heat‐killed pathogens, we added LPS. However, LPS alone failed to induce T‐cell cytokine production (Figure S5B). As an additional control, we analyzed CD4^+^T‐cell responses to antigenic peptides derived from *Candida albicans* (MP65), Aspergillus (SHMT) and from EBV. Consistent with the results obtained with heat‐killed Candida, we observed a switch from IL‐17 to IL‐10 production in AD‐HIES (Figure 5C; Figure S5C). Notably, patients with severe chronic mucocutaneous candidiasis (see Figure 3A) had a particularly high response to *C. albicans* . CCR6^−^T‐cells responses to fungi were in contrast inconsistent (Figure S5D). However, CCR6^−^T‐cells produced some IFN‐γ in response to EBV‐derived peptides in HDs and AD‐HIES patients (Figure S5E), indicating that virus‐specific Th1 responses were intact [13].

**FIGURE 5:**
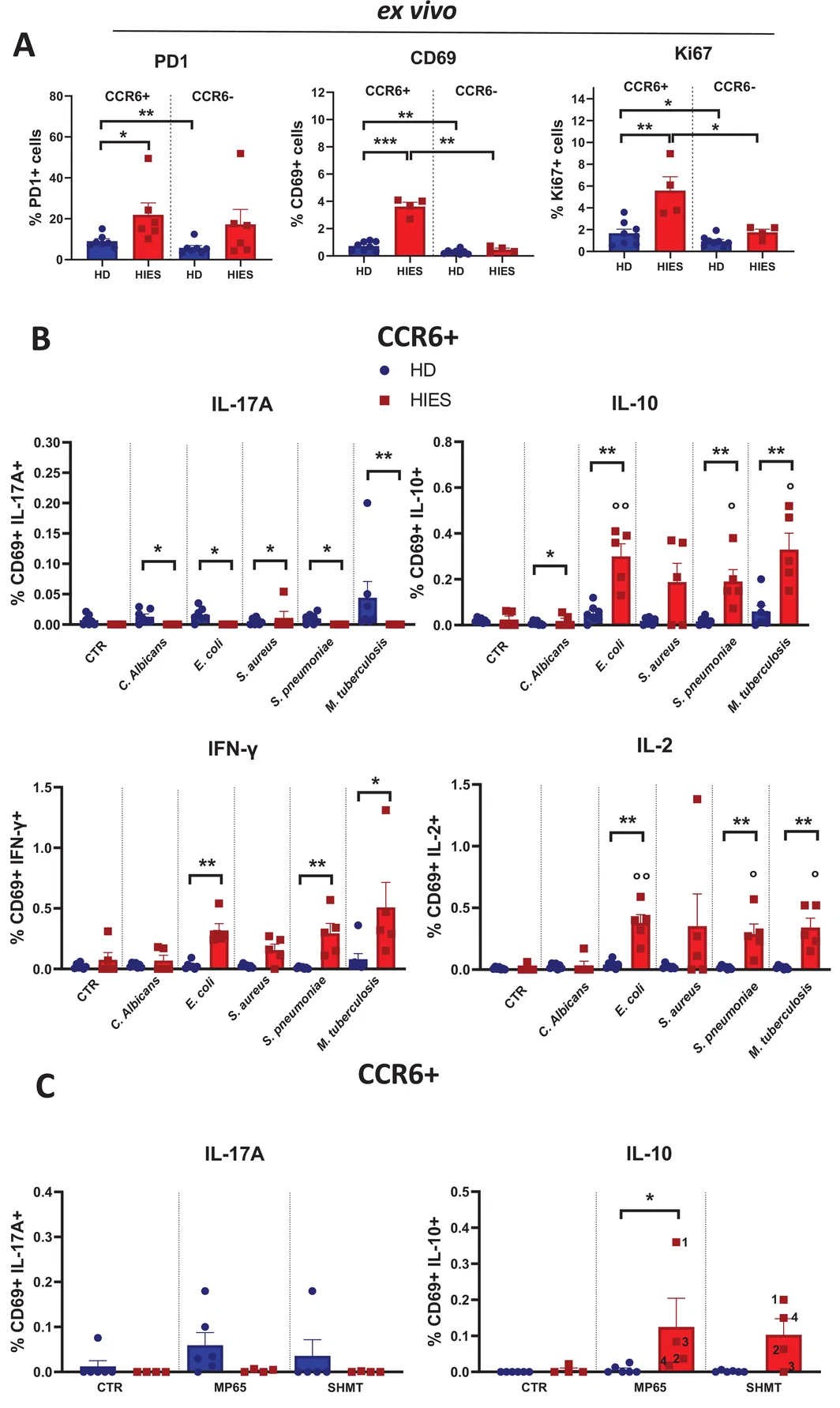
CCR6^+^T‐cells in AD‐HIES patients are activated in vivo and produce IL‐10 in response to opportunistic pathogens. (A) Ex vivo expression of PD1 (left), CD69, (center) and Ki67 (right) in gated CCR6^+^ and CCR6^−^T‐cells from AD‐HIES patients (red bars, *n* = 6) and HD (blue bars, *n* = 7). (B) Co‐expression of CD69 and IL‐17A (upper left panel), IL‐10 (upper right panel), IFN‐γ (lower left panel) and IL‐2 (lower right panel) in CD4^+^CCR6 T‐cells from AD‐HIES patients (red bars, *n* = 5) and HD (blue bars, *n* = 8) stimulated with the indicated heat‐killed pathogens. Cells incubated without pathogens were analyzed as negative control (CTR). (C) Co‐expression of CD69 and IL‐17A or IL‐10 in CCR6^+^T‐cells from AD‐HIES patients (red bars, *n* = 4) and HD (blue bars, *n* = 6) induced by antigenic peptide pools derived from *Candida albicans* (MP65) or from *Aspergillus fumigatus* (AMT). The patient numbers of Figure 3A are indicated for IL‐10^+^CCR6^+^T‐cells in AD‐HIES patients. Cells incubated without peptides were analyzed as negative control (CTR). Statistical significances were calculated with student's *t*‐test.

In summary, CCR6^+^T‐cells are specifically activated by bacteria and fungi, including those causing recurrent infections in AD‐HIES. However, they produce IL‐10 instead of IL‐17 in AD‐HIES patients, suggesting that pathogen‐specific CCR6^+^Th‐cells are arrested at a pre‐Th17 stage.

### 3.5 CCR6 ^+^T‐Cells in AD‐HIES Possess B‐Helper Functions and Promote IgE Production

CCR6^+^Th‐cells possess B‐helper functions in STAT3‐sufficient individuals and tonsillar CCR6^+^Th‐cells could induce some IgE [17]. We investigated here if they could induce antibody production also in patients with DN‐STAT3 mutations. Their B‐cell compartments were characterized by a strong decrease of memory cells [39], which was again compensated by an increase of naïve cells (Figure 6A; Figure S6A). However, they contained higher frequencies of plasmablasts, suggesting ongoing B‐cell activation (Figure 6B; Figure S6B). We co‐cultured autologous B‐ and Th‐cells in the presence of SEB and measured antibody production by ELISA. CCR6^+^Th‐cells from AD‐HIES patients induced significant amounts of IgG, demonstrating that they possessed B‐helper functions (Figure 6C). Notably, CXCR5^+^Tfh‐cells possessed comparable B‐helper capacities, whereas naïve CD4^+^T‐cells were inefficient. IgE production was undetectable under these conditions, presumably because CD4^+^T‐cells in the blood possess only low IL‐4 producing capacities (Figure S4D). We therefore performed optimized T‐B co‐cultures (see Material and Methods) in the presence of IL‐4. We also excluded CXCR3^+^T‐cells, because they lack B helper functions and produce IFN‐γ that may inhibit IgE [15, 40]. Significant amounts of IgE were induced by CCR6^+^Th‐cells from STAT3‐sufficient donors and from AD‐HIES patients under these conditions (Figure 6D). IgE and in particular IgG levels were however overall lower in AD‐HIES patients. Notably, IgE production induced by CCR6^+^T‐cells was in mean similar to that induced by Tfh2‐cells, which were analyzed as a positive control [15]. However, the relative potencies of CCR6^+^Th‐cells and Tfh2 to induce IgE and IgG were highly variable in individual donors (Figure 6E). Intriguingly, in two allergic donors B‐cells produced higher levels of IgE in co‐cultures with CCR6^+^Th‐cells than with Tfh2‐cells (Figure 6D,E). Finally, we analyzed if there was any correlation between serum IgE levels and CCR6^+^T‐cells in three AD‐HIES patients (Figure S6C). Interestingly, both Candida and Aspergillus‐specific IgE levels in the serum correlated with IL‐10 production induced by the relevant fungal antigens (Figure 5C).

**FIGURE 6:**
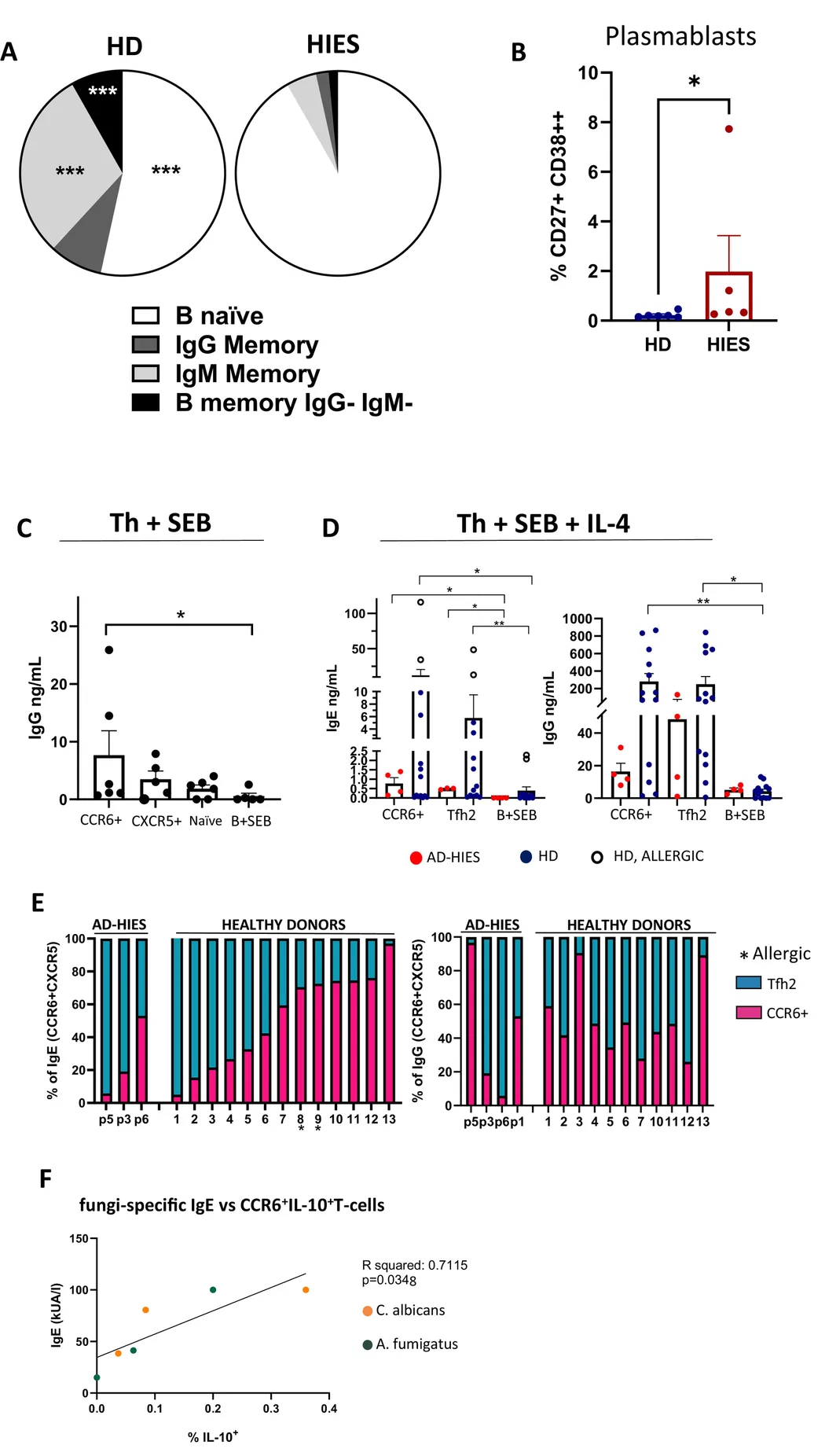
CCR6^+^T‐cells from AD‐HIES patients possess B helper capabilities and promote IgE production. (A) Pie Charts show the compositions of the B cell compartments in healthy donors (*n* = 6) and AD‐HIES patients (*n* = 5). (B) Frequencies of plasmablasts (CD19^+^CD27^hi^CD38^hi^) in HDs and AD‐HIES patients. (A, B) Statistical significances were calculated with an unpaired *t*‐test. (C) B‐cells from 6 ad‐HIES patients were cultured with SEB in the absence or presence of FACS‐purified CCR6^+^ or CXCR5^+^ Th‐cells and IgG secretion was assessed by ELISA. Statistical significance between was calculated by a Mann–Whitney test. (D) B‐cells from 13 healthy donors and 4 AD‐HIES patients analyzed in 7 experiments were cultured with SEB and IL‐4 in the absence or presence of CCR6^+^CXCR3^−^CXCR5^−^ Th‐cells and Tfh2‐cells and IgG and IgE secretion assessed by ELISA. IgE induction is also shown for two allergic donors (open circles). Statistical significances between B‐cells from STAT3‐sufficient donors or from AD‐HIES patients cultured with either SEB alone or in the presence of B helper T‐cells was calculated by a *t*‐test. When patients were analyzed twice the mean values are reported. (E) Relative potencies of CCR6^+^T‐cells and Tfh2 to induce IgG and IgE in STAT3‐sufficient donors and AD‐HIES patients. The sum of Igs induced by the two B helper subsets was set to 100% and the relative contributions of CCR6^+^Th‐cells versus Tfh2‐cells is shown in red and in blue, respectively. (F) *C. albicans* and *A. fumigatus* ‐specific IgE serum levels of AD‐HIES patients (Figure 3A) were plotted against the percentages of CCR6^+^T‐cells that produced Il‐10 in response to peptides derived from a relevant fungal antigen (Figure 5C). R squared and *p*‐values are reported in the panel.

Overall, these results show that CCR6^+^Th‐cells possess B‐helper functions in AD‐HIES patients and support IgE production by B cells with a similar efficiency as Tfh2 cells.

## 4 Discussion

We mapped here the differentiation stage of an enigmatic population of CCR6^+^B‐helper T‐cells that we identified previously [16, 17]. These CCR6^+^Th‐cells displayed a T_CM_ phenotype and expressed ROR‐γt, indicating that they represent an early stage of Th17 differentiation. Consistently, they acquired IL‐17 producing capacities upon TCR stimulation in the absence of exogenous Th17‐promoting cytokines. This “spontaneous” Th17 differentiation was fast and highly efficient. Conversely, Th17 differentiation of uncommitted human helper T‐cells is slow, inefficient and requires a combination of several Th17‐promoting cytokines (28). Thus, CCR6^SP^T‐cells are pre‐committed to a Th17 fate (“pre‐Th17 cells”), in analogy to T_CM_ subsets of CXCR3^+^pre‐Th1 and CCR4^+^pre‐Th2 that we have identified previously [41]. A feature of these pre‐committed T_CM_ is that they generate spontaneously effector cells of their respective lineage, but that they retain nevertheless a relevant degree of plasticity to differentiate to alternative fates upon instruction by polarizing cytokines [34]. In the case of pre‐Th17‐cells, IL‐12 induced IFN‐γ and promoted the generation of Th1/17‐cells in vitro. Consistently, AD‐HIES patients contained residual Th1/17‐cells that produced IFN‐γ in response to *M. tuberculosis* , explaining why *M. tuberculosis* is controlled in AD‐HIES in contrast to ROR‐γt‐deficient patients [42].

STAT3 signaling has a non‐redundant role in Th17 differentiation, as demonstrated by the absence of IL‐17 producing T‐cells in humans and mice with genetic defects in STAT3 activation [23, 25, 35, 43]. Naïve T‐cells developing into Th17‐cells activate STAT3 initially in response to IL‐6, which then induces IL‐21 production to amplify STAT3 activation and Th17 differentiation in an autocrine feed‐forward loop [18, 44, 45]. Consistently, Th17 differentiation of pre‐Th17‐cells was inhibited when STAT3‐activating cytokines were neutralized in vitro or when STAT3 signaling was impaired in vivo, i.e., in AD‐HIES. Notably, TGF‐β induces CCR6 and ROR‐γt in human CD4^+^T‐cells but inhibits IL‐17 production [16, 46]. Once induced, CCR6 expression in human CD4^+^T‐cells is remarkably stable [47]. Thus, pre‐Th17‐cells may be generated with TGF‐β, which also induces CCR4 [48], CXCL13 [49] and IL‐10 [44], in an at least partially STAT3‐independent manner.

STAT3 signaling is also critical for Tfh differentiation. We observed a selective lack of Tfh17‐cells in AD‐HIES. Tfh1/2 subsets were in contrast present, indicating distinct, STAT3‐independent differentiation requirements. Importantly, AD‐HIES patients contained residual CCR6^+^Th‐cells that possessed B‐helper functions, since they could induce IgG and IgE from autologous B‐cells. These CCR6^+^Th‐cells were activated by opportunistic pathogens to produce IL‐10, which promotes IgG production in STAT3‐sufficient individuals [17]. Intriguingly, fungi‐specific CCR6^+^T‐cells correlated with fungi‐specific IgE serum levels. With the caveat of the low patient numbers, these findings suggest a causal link between antigen‐specific CCR6^+^T‐cells and IgE. Ig levels in T‐B co‐cultures from AD‐HIES were overall low, consistent with a role for IL‐21/STAT3 not only in IgG but also in IgE production [50, 51]. However, the pro‐survival effects of STAT3 signaling in lymphocytes may also contribute [52]. Interestingly, CCR6^+^Th‐cells and Tfh2 induced in mean similar amounts of IgE. It is thus tempting to speculate that CCR6^+^Th‐cells may contribute to IgE production not only in HIES, but also in allergy. Consistently, CCR6^+^Th‐cells from two STAT3‐sufficient allergic donors induced very high levels of IgE. If pre‐Th17‐cells react with allergens remains however to be investigated [53]. Notably, CCR6^+^Th‐cells are extrafollicular B‐helper T‐cells [17] and may thus induce antibodies of lower quality. Evidence for extrafollicular IgE induction was reported both in humans [54] and mice [51]. Consistent with a role for extrafollicular helper T‐cells in aberrant IgE induction in AD‐HIES, IgE from patients were reported to contain reduced somatic hypermutations [39].

Inborn errors of immunity unveil non‐redundant pathways for the immune defense against pathogens. AD‐HIES patients lack Th17‐cells and experience consequently recurrent infections with extracellular pathogens [26, 55]. These opportunistic pathogens activated selectively the residual CCR6^+^T‐cells to produce IL‐10, but also IL‐2 and IFN‐γ. IL‐10 producing Th17‐cells specific for *S. aureus* have previously been described in healthy individuals [6], but they lacked protective functions in mice [56]. Notably, since IL‐10 activates STAT3 it is probably functionally irrelevant in AD‐HIES [57]. The increased IL‐10 production by CCR6^+^T‐cells in AD‐HIES patients was nevertheless surprising, since IL‐10 production in developing Th17‐cells is promoted by IL‐6 and IL‐21 that signal via STAT3 [34, 44]. Moreover, IL‐21 failed to induce IL‐10 production by in vitro activated T‐cells from AD‐HIES patients [58]. Our results indicate however that STAT3 does not play a non‐redundant role for T‐cell IL‐10 production in vivo, neither by helper nor by regulatory T‐cells. Indeed, T‐cell IL‐10 production can also be induced by IL‐4 or IL‐12 via STAT4 or STAT6, respectively [59], or by chronic TCR stimulation. Since CCR6^+^Th‐cells were activated by pathogens that cause recurrent infections in AD‐HIES patients, their increased IL‐10 production is probably due to chronic TCR stimulation. Consistently, patients with severe chronic mucocutaneous candidiasis contained relatively high levels of Candida‐specific IL‐10 producing CCR6^+^T‐cells.

In conclusion, AD‐HIES‐associated pathogens induce the generation of pre‐Th17‐cells that produce IL‐10. The latter cannot differentiate to Th17‐cells in the absence of STAT3 signaling and consequently fail to control infections with AD‐HIES‐associated pathogens, but they possess B helper functions and may thus contribute to aberrant IgE production (Graphical Abstract).

## Author Contributions

G.M. and C.V. designed experiments, performed and supervised experiments, analyzed the data and wrote the paper. F.C. performed bioinformatic analysis. P.L. S.M., E.S., E.C., N.P., M.L.S., M.C., M.B.I. analyzed and/or performed experiments. L.R. and S.A. provided critical Discussion and wrote the paper. L.A.B., R.M.D., M.C. and G.F. provided access to patients and their clinical characteristics and wrote the paper. J.G. designed the study, supervised experiments and wrote the paper.

## Funding

This work was supported by Fondazione Telethon and Ministero dell'Università e della Ricerca.

## Conflicts of Interest

The authors declare no conflicts of interest.

## Supporting information


**Figure S1:** (A) Gating strategy to identify CCR6^+^Th subsets in human blood (B)/(C) Frequencies of, Integrin‐β7^+^, CXCR6^+^, CLA^+^, Integrin‐β1^+^, CCR4^+^, CD27^+^ and PD1^+^ cells were measured ex vivo by surface staining on gated CCR6^+^T‐cell subsets. (D) Stacked histograms show co‐expression of TCF7/LEF1 in CD4^+^ naïve (CD45RA^+^CCR7^+^CD95^−^), T_SCM_ (CD45RA^+^CCR7^+^CD95^+^), T_CM_ and T_EM_. (E) RORγt expression in CCR6^+/−^ naïve (CCR7^+^CD45RA^+^), central (CCR7^+^CD45RA^−^, T_CM_) and effector memory (CCR7^−^CD45RA^−^, T_EM_) T‐cells from healthy donors (*n* = 8). Statistical significances were calculated using One way ANOVA. (F) Immunofluorescence for IL‐10 production in Tfh17‐cells following stimulation with monocytes and SEB for 16 h. (G) Negative controls for STAT1/3 activation in the absence of recombinant IL‐6 and IL‐23.
**Figure S2:** (A) Experimental strategy to analyze Th17 differentiation of non‐polarized CCR6^SP^T‐cells. (B) FACS‐purified CCR6^SP^‐T‐cells were stimulated with anti‐CD3/CD28‐coated beads in the absence or presence of recombinant TGF‐β, WNT10A, IL‐4 or IL‐12 as indicated. After 6 days cells were restimulated with PMA and Ionomycin and production of IL‐22 analyzed. Statistical significance was calculated by One‐way ANOVA. (**C**) FACS dot plots of one representative experiment showing IL‐17 versus IFN‐γ production by CCR6^SP^‐T‐cells stimulated with anti‐CD3/CD28‐coated beads in the absence or presence of IL‐4 or IL‐12. (**D**) The day 6 culture supernatant of anti‐CD3/CD28‐stimulated CCR6^+^‐T‐cell subsets was analyzed for IL‐6 by ELISA (*n* = 3). (**E**) Dot plots of one representative experiment showing CCR6^SP^T‐cells cultured with anti‐CD3/28‐coated beads in the absence or presence of anti‐IL‐6 and anti‐10/10R antibodies and soluble IL21R(s21R) were analyzed for IL‐17 and IL‐22 production.
**Figure S3:** (A) Heatmap of median normalized expression of markers within each of the identified 16 clusters. Dendrogram on the right side of the heatmap shows hierarchical clustering among the identified clusters. Red indicates high and blue low expression. (B) Histograms showing the expression levels of the 13 markers in the 16 CD4^+^T‐cell clusters. (C) UMAPs showing the expression of the individual 13 markers. Red indicates high, green intermediate and blue low expression levels. (D) Gating strategy.
**Figure S4:** (A) Frequencies of naïve (CD45RA^+^CCR7^+^CD95^−^) and memory T‐cell subsets, i.e., Tscm (CD45RA^+^CCR7^+^CD95^+^), Tcm (CCR7^+^CD45RA^−^), Tem (CCR7^−^CD45RA^−^) and Temra (CCR7^−^CD45RA^+^) in HDs (blue bars) and AD‐HIES patients (red bars). (B) Frequencies of Th1‐ (CD4^+^CXCR3^+^CCR4^−^CCR6^−^) and Th2‐cells (CD4^+^CXCR3^−^CCR4^+^CCR6^+^), as well as Tregs (CD4^+^FOXP3^+^IL‐7R^lo^) and Tr1‐cells (CD4^+^GZMK^+^IL‐7R^lo^) (36) in HDs (blue bars, *n* = 7) and AD‐HIES patients (red bars, *n* = 6). (C) Expression of β7‐integrin, CLA and CCR10 in gated CCR6^+^ and CCR6^−^Th‐cells in HD and AD‐HIES patients. (D) Production of the indicated cytokines by ex vivo stimulated CCR6^+^ and CCR6^−^ T‐cell subsets of healthy donors (HD, blue, *n* = 7) and AD‐HIES patients (red, *n* = 4). (E) Left: Frequencies of IL‐10‐producing cells after 30 h stimulation with anti‐CD3 antibodies and IL‐2 of CD4^+^T‐cell subsets sorted according to CCR6 expression from AD‐HIES patients (red bars, *n* = 4) and healthy donors (blue bars, *n* = 6). Right: Frequencies of IL‐10 producing cells among superantigen (SEB)‐stimulated, gated CCR6^+^ and CCR6^−^T‐cells from AD‐HIES patients (red bars, *n* = 5) and HD (blue bars, *n* = 7). (F) Representative Dot plots showing intracellular IL‐10 and IFN‐g staining of ex vivo–stimulated T regulatory subsets from healthy donors (left) AD‐HIES patients (right).
**Figure S5:** (A) Co‐expression of CD69 and the indicated cytokines in CCR6^−^T‐cells from AD‐HIES patients (red bars, *n* = 4–5) and healthy donors (HDs, blue bars, *n* = 7–8) upon stimulation with pathogen‐derived antigens. (B) Co‐expression of CD69 and the indicated cytokines in CCR6^+^ (upper panels) and CCR6^−^ (lower panels) T‐cells from AD‐HIES patients (red bars, *n* = 4–5) and healthy donors (HDs, blue bars, *n* = 7–8) upon stimulation with LPS. Unstimulated cells were analyzed as negative control (“Unstim”) and SEB as positive control. (C) CCR6^+^ or (D) CCR6^−^T‐cells were stimulated with antigenic peptide pools derived from *C. albicans* (MP65) or A. fumigatus (SHMT) and the co‐expression of CD69 and the indicated cytokines analyzed by flow cytometry. (E) Co‐expression of CD69 and IFN‐γ in CCR6^−^ Th‐cells from AD‐HIES patients (red bars, *n* = 4–5) and healthy donors (HDs, blue bars, *n* = 8) stimulated with an EBV‐derived peptide pool. Statistical significances between HDs and AD‐HIES were calculated with unpaired *t*‐tests.
**Figure S6:** Gating strategies for (A) B‐cell subsets and (B) plasmablasts.
**Supplementary Methods**. Immunofluorecscence.
Cells were fixed in 4% paraformaldehyde (PFA) for 10 min at room temperature, washed with PBS and blocking buffer (10% Fetal Calf Serum in PBS) was added for 1 h at room temperature. Cells were then incubated with an anti‐human CCR6 mouse antibody (1:50, R&D Systems) and an anti‐CD4 rabbit monoclonal antibody (1:30, Abcam) overnight at +4°C. Subsequently, cells were washed and incubated with a chicken anti‐mouse IgG Alexa Fluor 568 (1:1000, ThermoFisher Scientific) and a goat anti‐rabbit IgG Alexa Fluor 488 (1: 1000, ThermoFisher Scientific) for 1 h at room temperature. Cells were washed with PBS, permeabilized with 0.1% Triton X‐100 for 15 min, and incubated overnight with a rat anti‐human IL‐10 antibody (1:100, ThermoFisher, USA). Cells were washed with PBS and incubated with a donkey anti‐rat IgG Alexa Fluor 647 secondary antibody for 1 h at room temperature. After washing with PBS, cells were counterstained with DAPI (Molecular Probes, ThermoFisher Scientific) and mounted using ProLong Diamond and ProLong Glass mounting reagents (ThermoFisher Scientific). Labelling control (no secondary antibody) was performed before starting the experiments. The isotype control was included in each experiment.

**Table S1:** List of antibodies.

**Table S2:** List of Antigens.

## Data Availability

The data that supports the findings of this study are available in the Supporting Information of this article.
