# Supplementary material for: Autosomal Dominant Hyper‐IgE Syndrome Patients Retain IL10‐Producing preTh17‐Cells That Are Activated by Opportunistic Pathogens and Support IgE Production
Source: Allergy. 2026 Feb 25;81(11):3864–79. doi: 10.1111/all.70266 (PMC13753684; doi:10.1111/all.70266)

sFigure 1

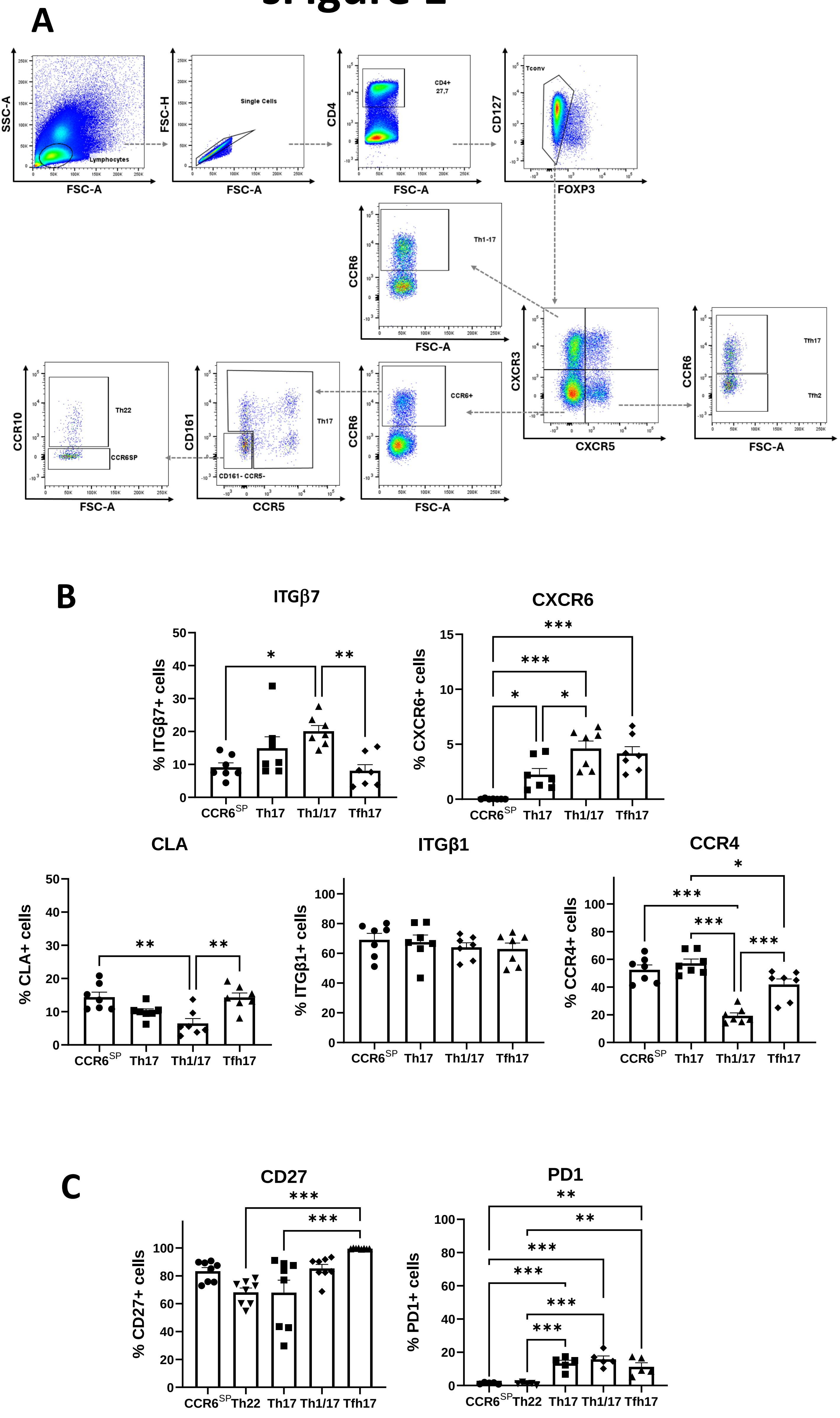
A
SSC-A
FSC-A
Lymphocytes
FSC-H
Single Cells
CD4
CD4+
27,7
CD127
FOXP3
Tconv
CCR6
Th1-17
CXCR3
CXCR5
Tfh17
Tfh2
CCR6+
CD161
CCR5
Th17
CD161- CCR5-
CCR10
Th22
CCR6SP
B
ITGβ7
% ITGβ7+ cells
CXCR6
% CXCR6+ cells
CLA
% CLA+ cells
ITGβ1
% ITGβ1+ cells
CCR4
% CCR4+ cells
CCR6SP
Th17
Th1/17
Tfh17
C
CD27
% CD27+ cells
PD1
% PD1+ cells
CCR6SP
Th22
Th17
Th1/17
Tfh17

D

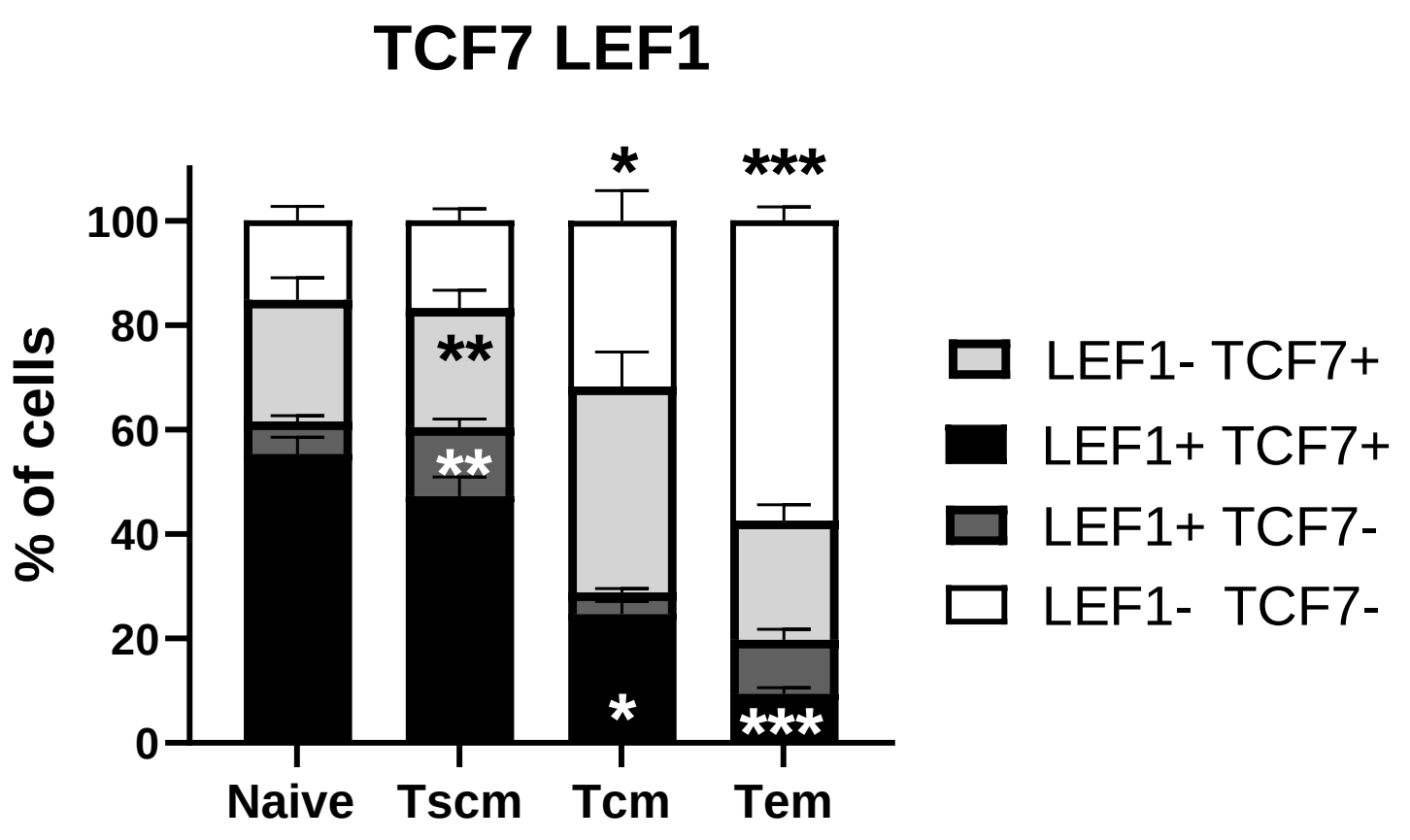
TCF7 LEF1
% of cells
100
80
60
40
20
0
*
***
**
**
*
***
Naive
Tscm
Tcm
Tem
LEF1- TCF7+
LEF1+ TCF7+
LEF1+ TCF7-
LEF1- TCF7-

E

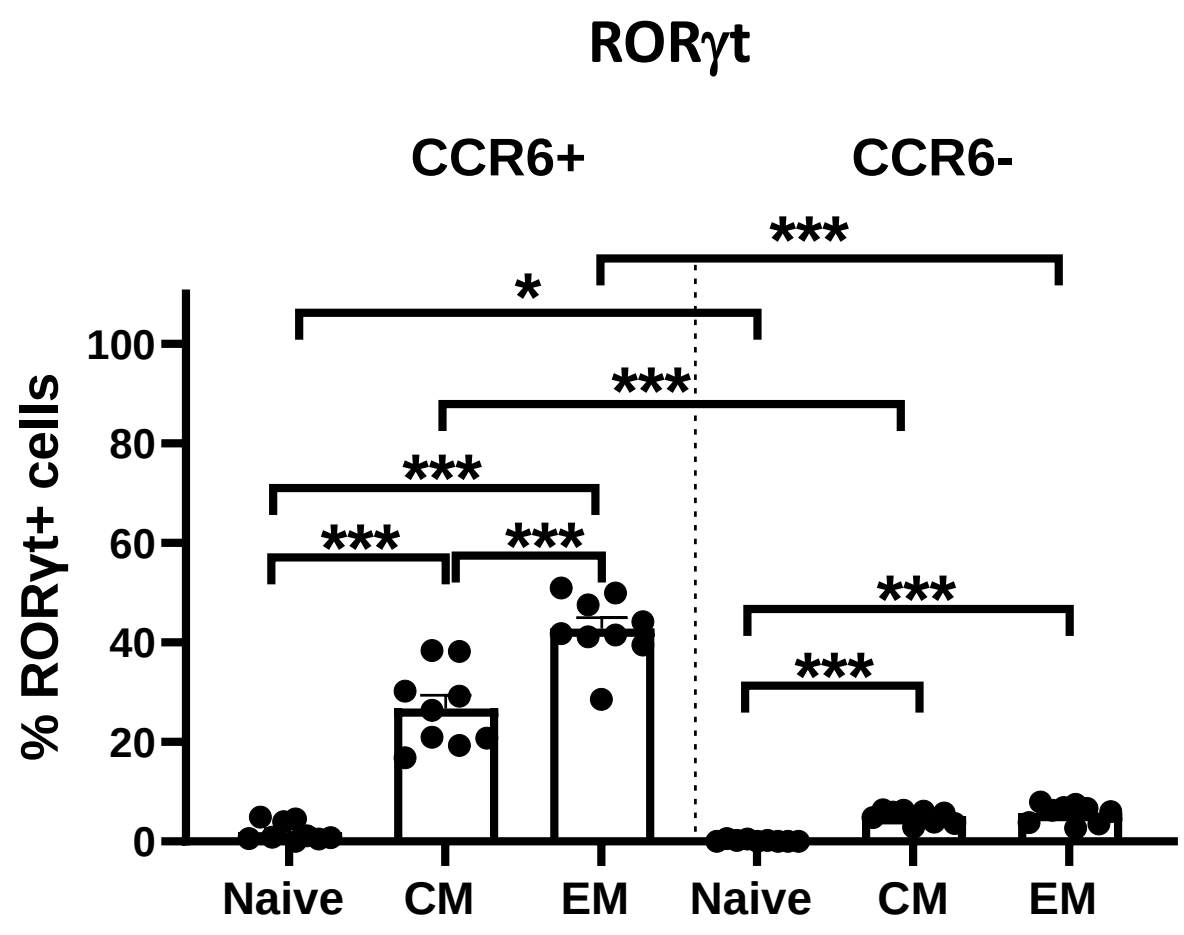
RORγt
CCR6+
CCR6-
% RORγt+ cells
100
80
60
40
20
0
*
***
***
***
***
***
***
***
Naive
CM
EM
Naive
CM
EM

F

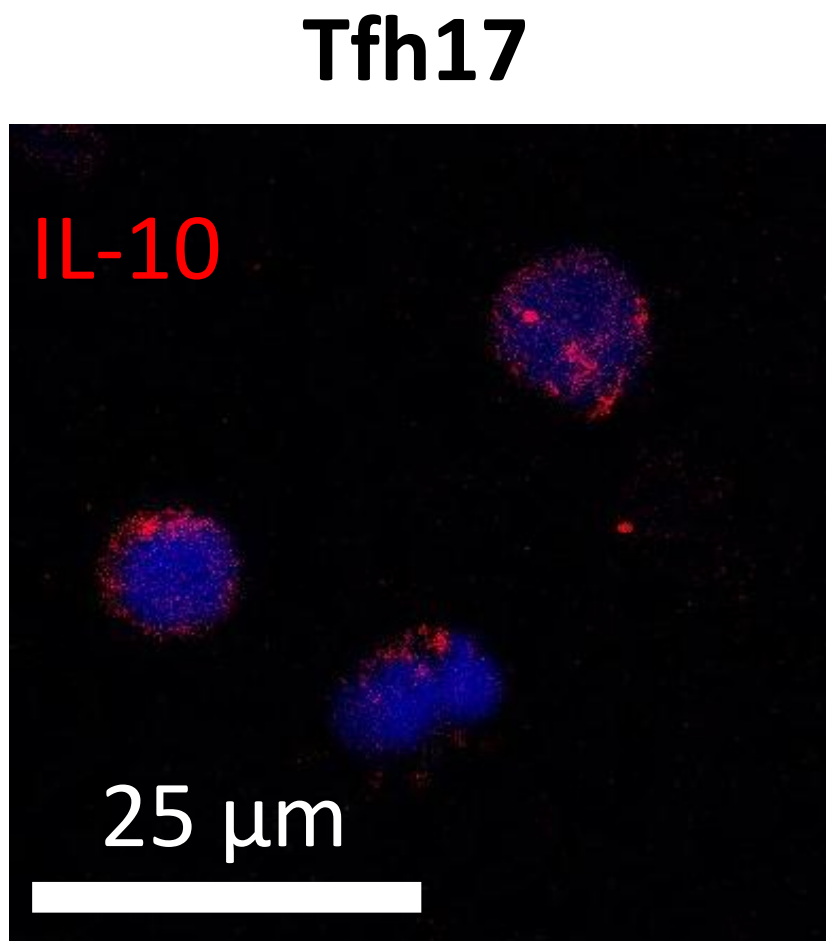
Tfh17
IL-10
25 µm

G

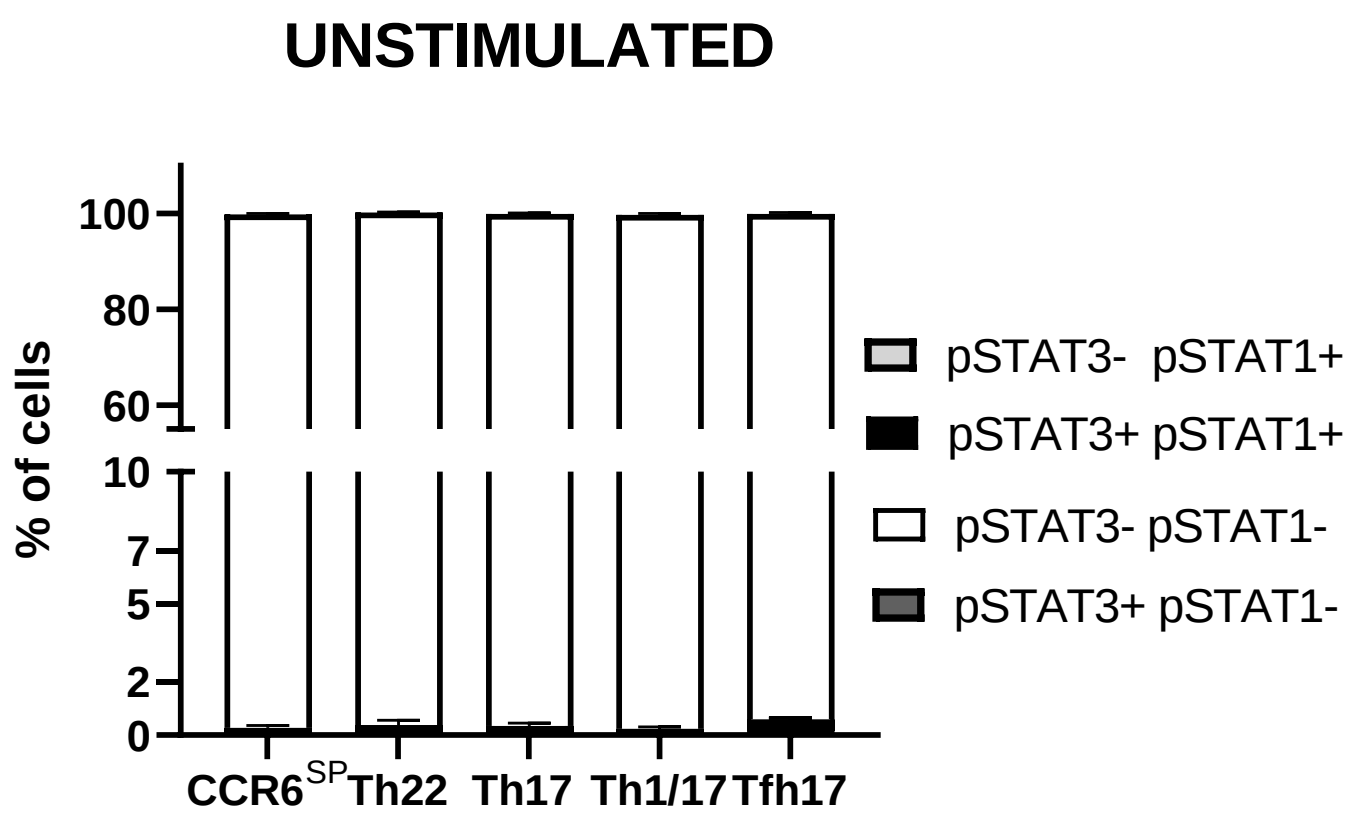
UNSTIMULATED
% of cells
100
80
60
10
7
5
2
0
CCR6SP
Th22
Th17
Th1/17
Tfh17
pSTAT3- pSTAT1+
pSTAT3+ pSTAT1+
pSTAT3- pSTAT1-
pSTAT3+ pSTAT1-

sFigure 2

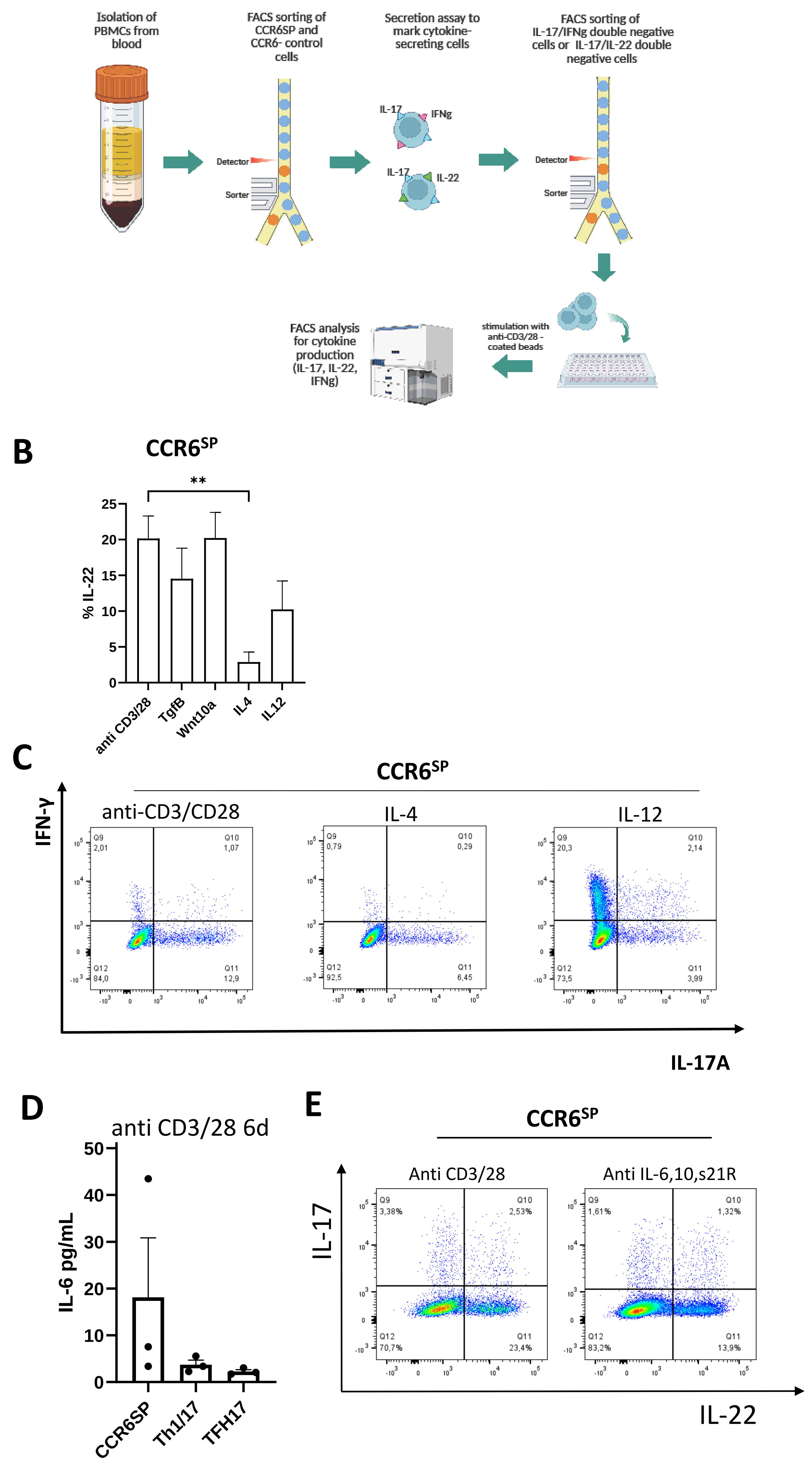
A
Isolation of PBMCs from blood
FACS sorting of CCR6SP and CCR6- control cells
Detector
Sorter
Secretion assay to mark cytokine-secreting cells
IL-17
IFNg
IL-17
IL-22
FACS sorting of IL-17/IFNg double negative cells or IL-17/IL-22 double negative cells
Detector
Sorter
stimulation with anti-CD3/28 - coated beads
FACS analysis for cytokine production (IL-17, IL-22, IFNg)
B
CCR6SP
**
% IL-22
anti CD3/28
TgfB
Wnt10a
IL4
IL12
C
CCR6SP
IFN-γ
anti-CD3/CD28
IL-4
IL-12
Q9 2,01
Q10 1,07
Q12 84,0
Q11 12,9
Q9 0,79
Q10 0,29
Q12 92,5
Q11 6,45
Q9 20,3
Q10 2,14
Q12 73,5
Q11 3,99
IL-17A
D
anti CD3/28 6d
IL-6 pg/mL
CCR6SP
Th1/17
TFH17
E
CCR6SP
Anti CD3/28
Anti IL-6,10,s21R
IL-17
Q9 3,38%
Q10 2,53%
Q12 70,7%
Q11 23,4%
Q9 1,61%
Q10 1,32%
Q12 83,2%
Q11 13,9%
IL-22

sFigure 3

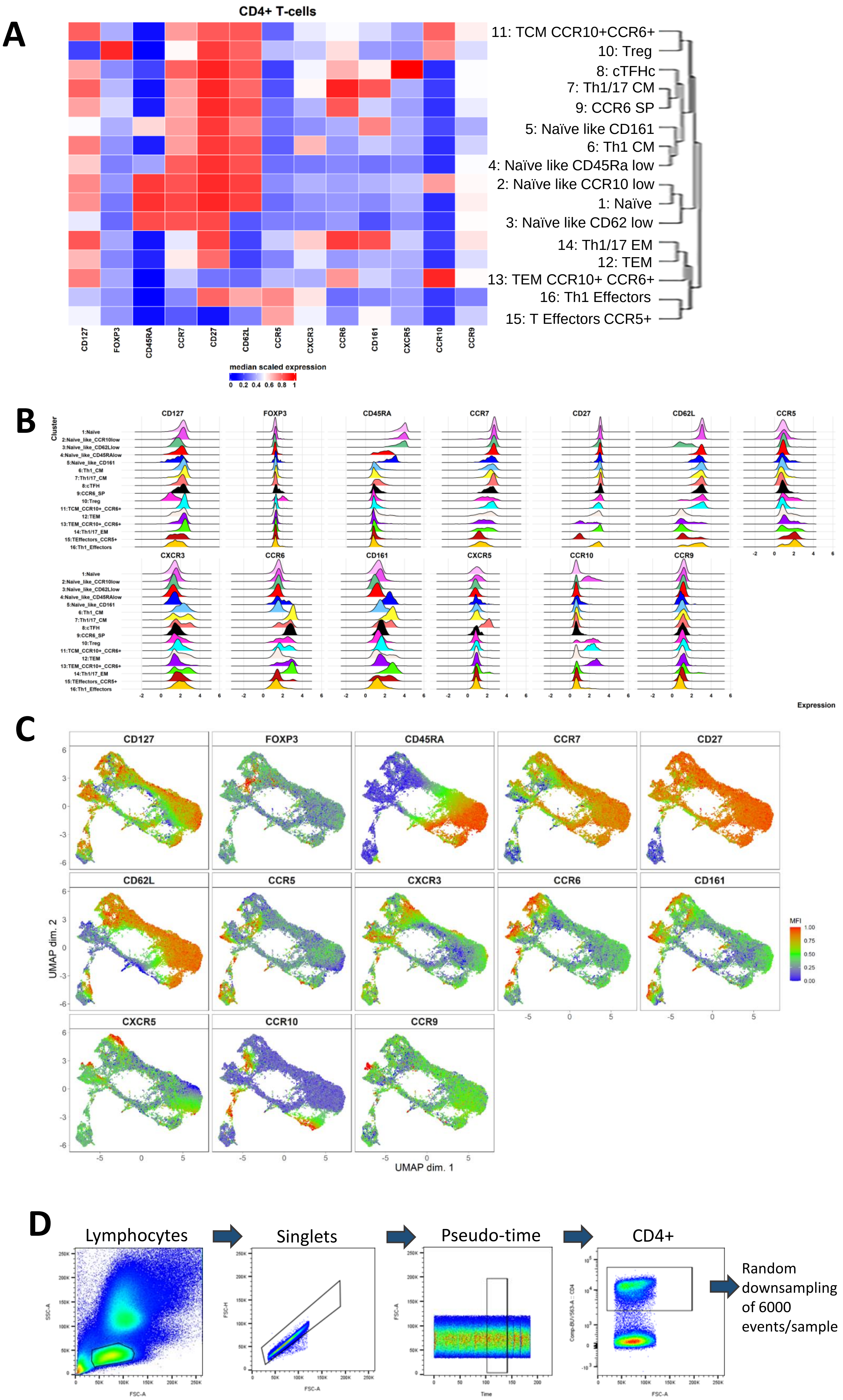
A
CD4+ T-cells
11: TCM CCR10+CCR6+
10: Treg
8: cTFHc
7: Th1/17 CM
9: CCR6 SP
5: Naïve like CD161
6: Th1 CM
4: Naïve like CD45Ra low
2: Naïve like CCR10 low
1: Naïve
3: Naïve like CD62 low
14: Th1/17 EM
12: TEM
13: TEM CCR10+ CCR6+
16: Th1 Effectors
15: T Effectors CCR5+
CD127
FOXP3
CD45RA
CCR7
CD27
CD62L
CCR5
CXCR3
CCR6
CD161
CXCR5
CCR10
CCR9
median scaled expression
B
Cluster
Expression
C
UMAP dim. 2
UMAP dim. 1
MFI
D
Lymphocytes
Singlets
Pseudo-time
CD4+
Random downsampling of 6000 events/sample

sFigure 4

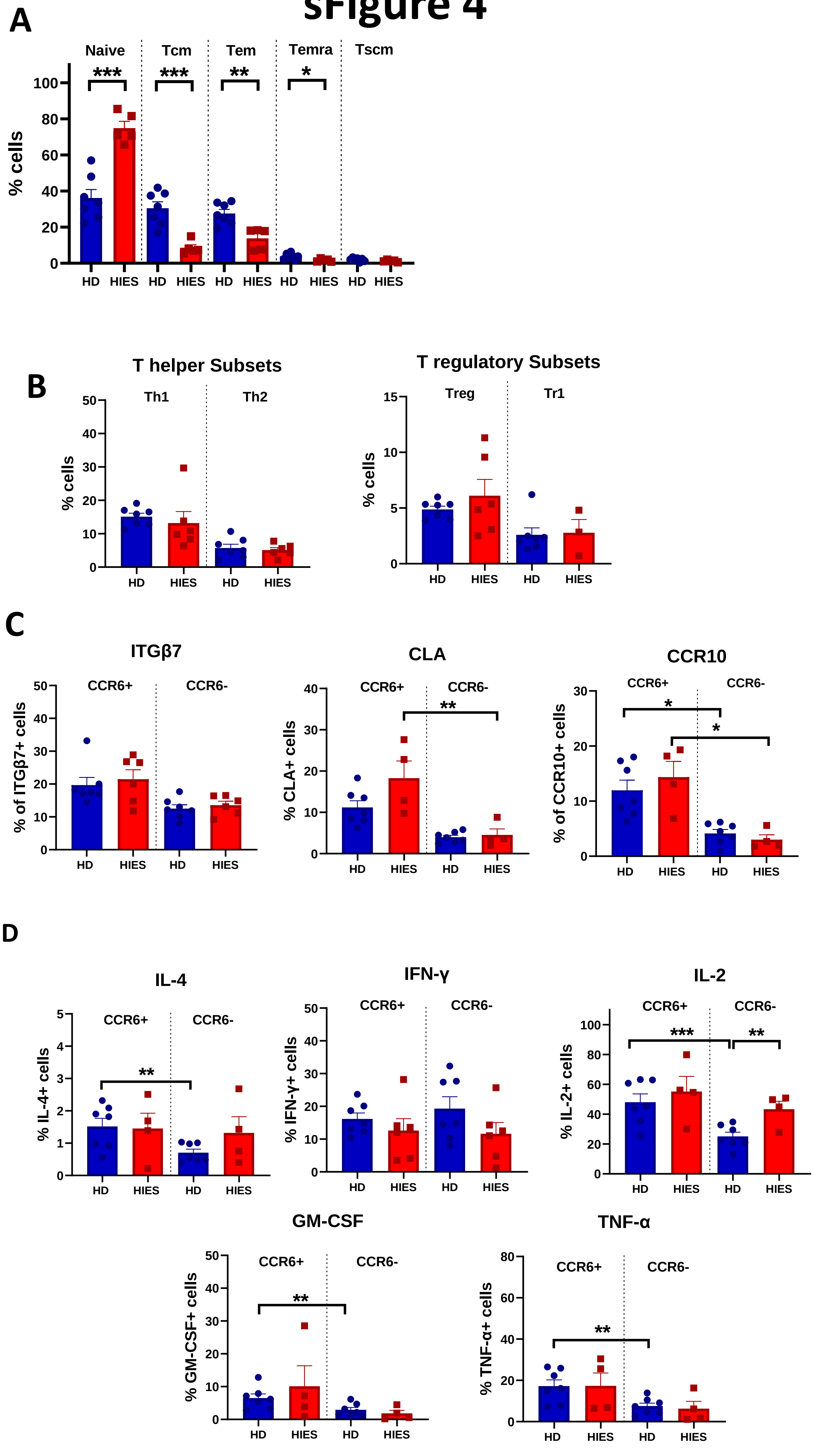
A
Naive
Tcm
Tem
Temra
Tscm
***
***
**
*
% cells
0
20
40
60
80
100
HD
HIES
HD
HIES
HD
HIES
HD
HIES
HD
HIES
B
T helper Subsets
Th1
Th2
% cells
0
10
20
30
40
50
HD
HIES
HD
HIES
T regulatory Subsets
Treg
Tr1
% cells
0
5
10
15
HD
HIES
HD
HIES
C
ITGβ7
CCR6+
CCR6-
% of ITGβ7+ cells
0
10
20
30
40
50
HD
HIES
HD
HIES
CLA
CCR6+
CCR6-
**
% CLA+ cells
0
10
20
30
40
HD
HIES
HD
HIES
CCR10
CCR6+
CCR6-
*
*
% of CCR10+ cells
0
10
20
30
HD
HIES
HD
HIES
D
IL-4
CCR6+
CCR6-
**
% IL-4+ cells
0
1
2
3
4
5
HD
HIES
HD
HIES
IFN-γ
CCR6+
CCR6-
% IFN-γ+ cells
0
10
20
30
40
50
HD
HIES
HD
HIES
IL-2
CCR6+
CCR6-
***
**
% IL-2+ cells
0
20
40
60
80
100
HD
HIES
HD
HIES
GM-CSF
CCR6+
CCR6-
**
% GM-CSF+ cells
0
10
20
30
40
50
HD
HIES
HD
HIES
TNF-α
CCR6+
CCR6-
**
% TNF-α+ cells
0
20
40
60
80
HD
HIES
HD
HIES

sFigure 4

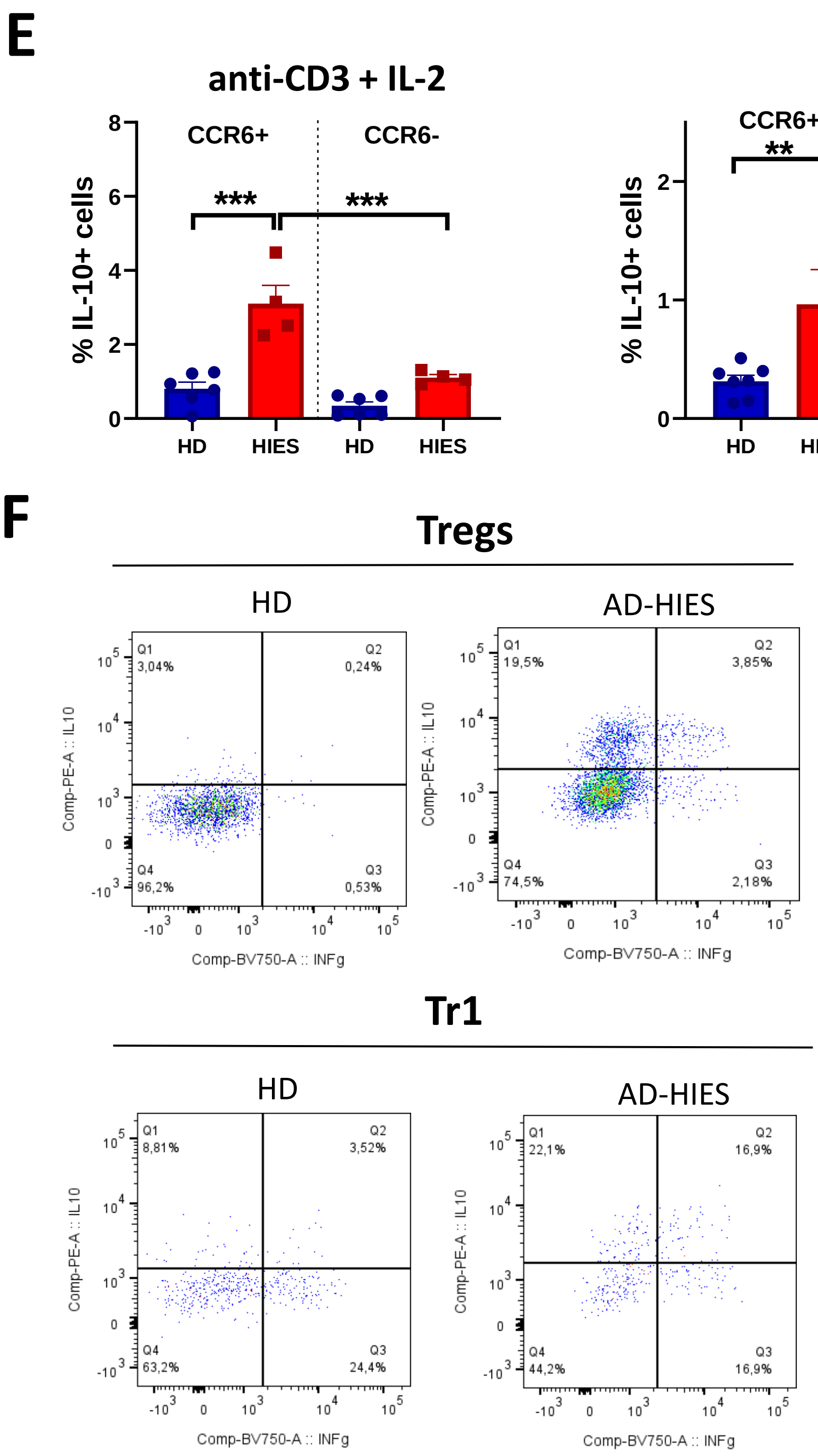
E
anti-CD3 + IL-2
CCR6+
CCR6-
***
***
% IL-10+ cells
0
2
4
6
8
HD
HIES
HD
HIES
SEB
CCR6+
CCR6-
**
**
% IL-10+ cells
0
1
2
HD
HIES
HD
HIES
F
Tregs
HD
AD-HIES
Q1
3,04%
Q2
0,24%
Q3
0,53%
Q4
96,2%
Q1
19,5%
Q2
3,85%
Q3
2,18%
Q4
74,5%
Comp-PE-A :: IL10
Comp-BV750-A :: INFg
Tr1
HD
AD-HIES
Q1
8,81%
Q2
3,52%
Q3
24,4%
Q4
63,2%
Q1
22,1%
Q2
16,9%
Q3
16,9%
Q4
44,2%
Comp-PE-A :: IL10
Comp-BV750-A :: INFg

sFigure 5

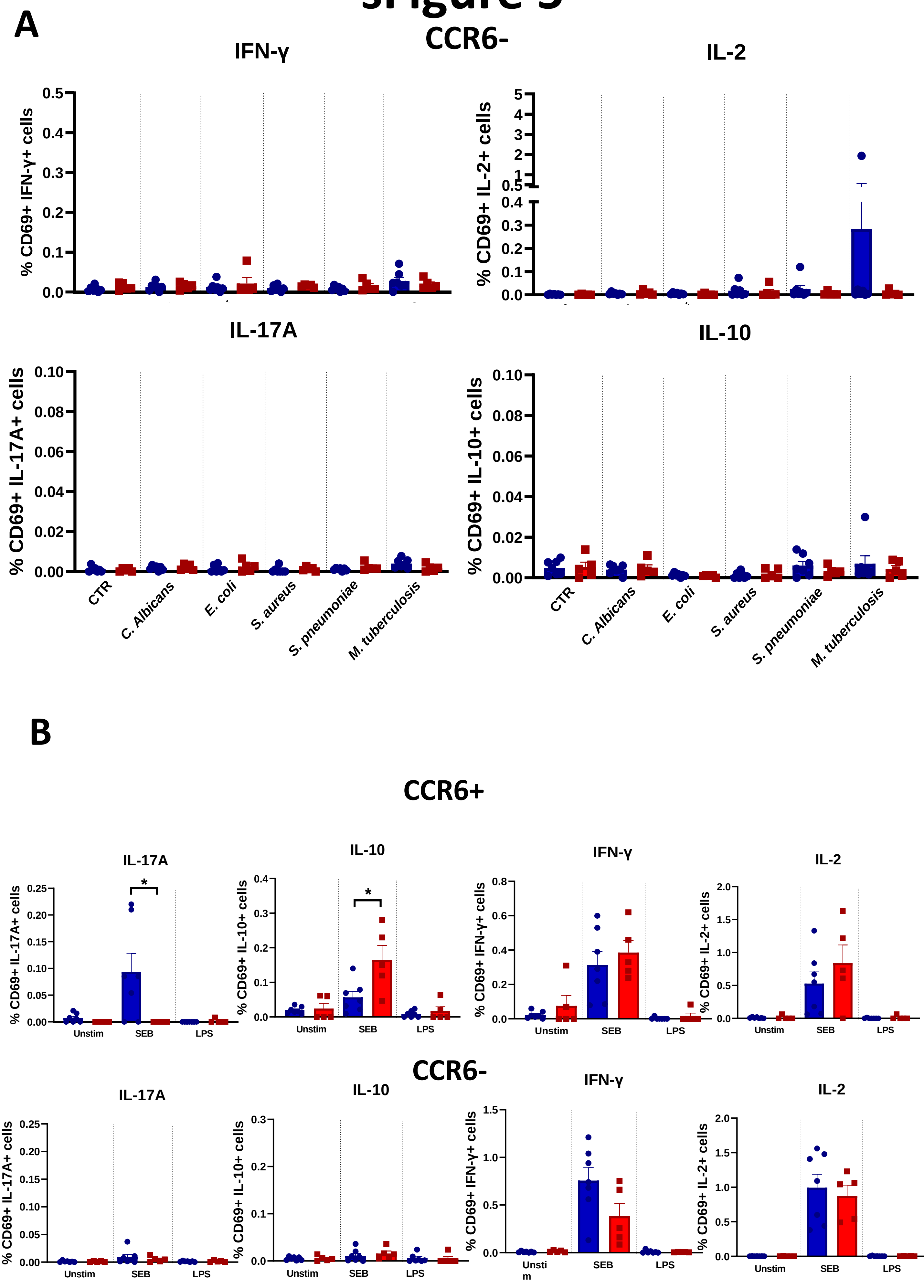
A
CCR6-
IFN-γ
IL-2
IL-17A
IL-10
% CD69+ IFN-γ+ cells
% CD69+ IL-2+ cells
% CD69+ IL-17A+ cells
% CD69+ IL-10+ cells
CTR
C. Albicans
E. coli
S. aureus
S. pneumoniae
M. tuberculosis
B
CCR6+
CCR6-
*
Unstim
SEB
LPS

sFigure 5

C

CCR6+

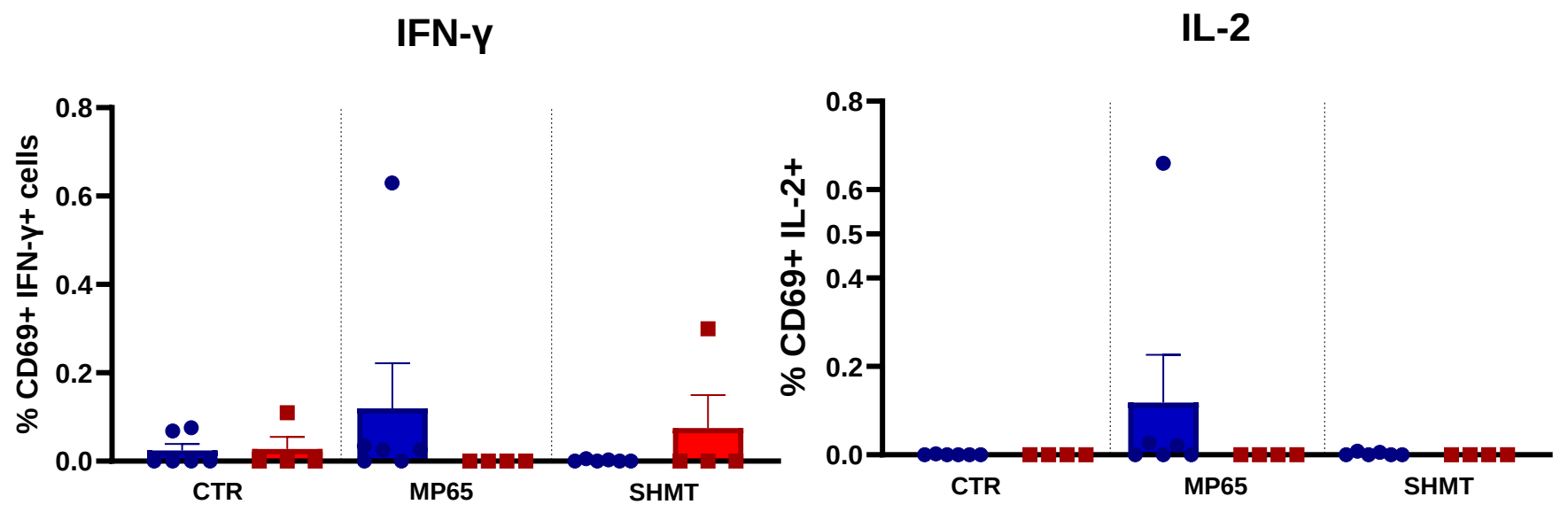

D

CCR6-

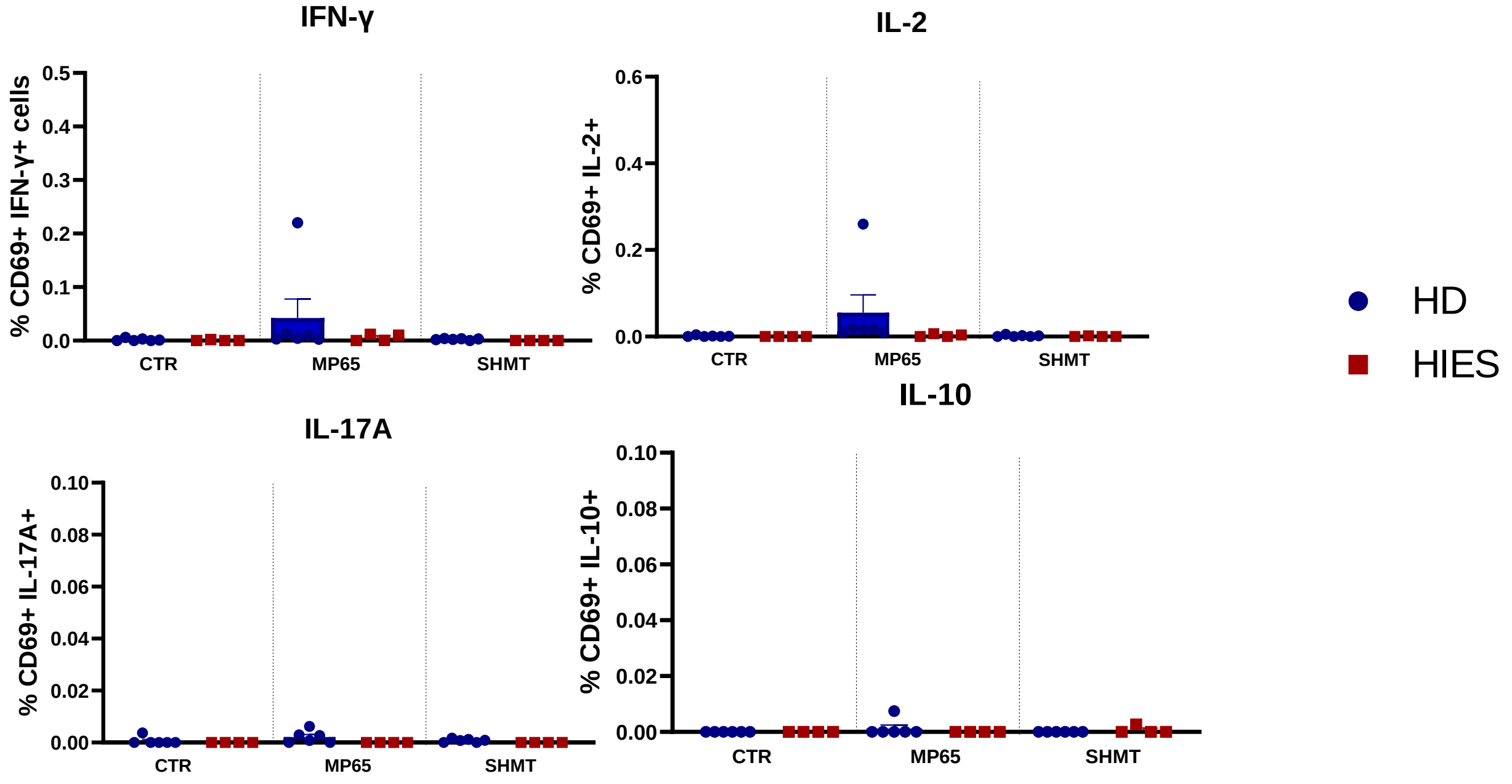

E

CCR6-

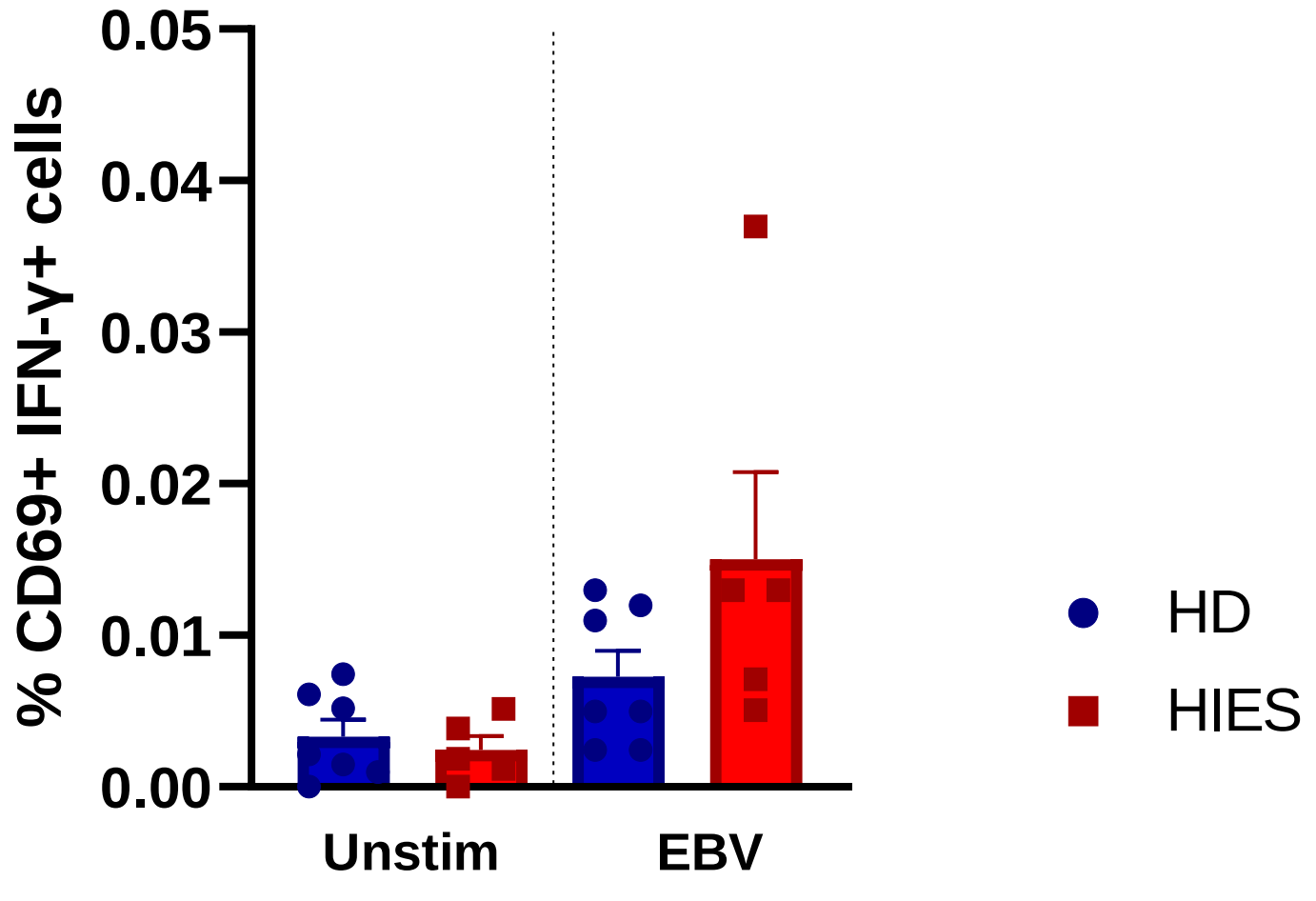

sFigure 6

A

B cells

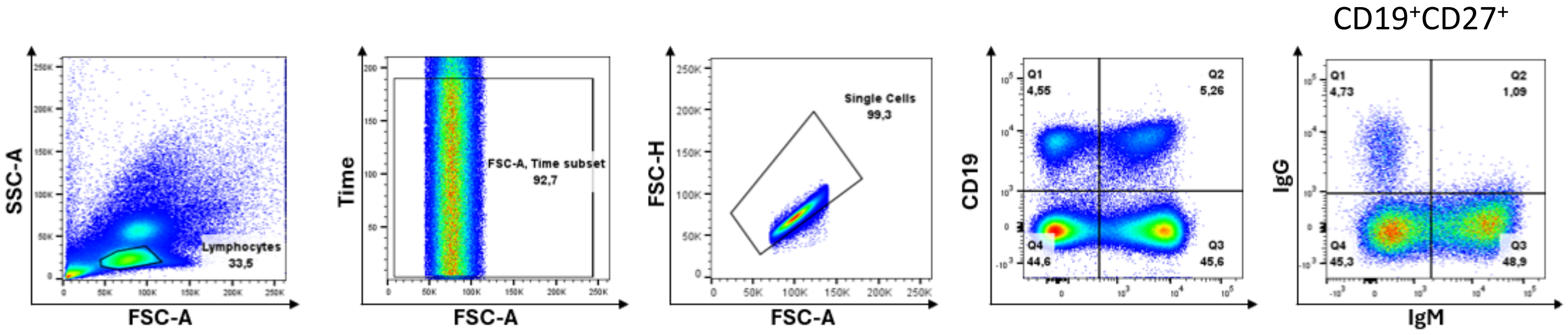

B

Plasmablasts

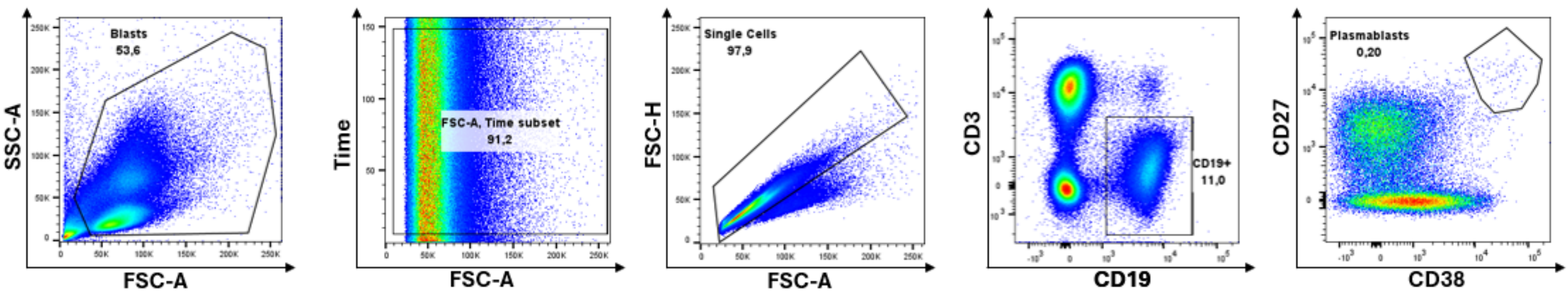

Supplement: Supplementary file 1 [file ALL-81-3864-s002.pdf]
